# Reduced Susceptibility to Cefiderocol Among Clinical MCR-1-Producing *Escherichia coli* Isolates from Tunisia

**DOI:** 10.3390/antibiotics15080802

**Published:** 2026-08-18

**Authors:** Nadia Jaidane, Thierry Naas, Souad Fayad, Pierre Châtre, Wejdene Mansour, Aymen Bouaziz, Pauline François, Laetitia Du Fraysseix, Bogdan I. Iorga, Nahed A. Al Laham, Lamia Tilouche, Farouk Barguellil, Marisa Haenni

**Affiliations:** 1 Laboratory of Metabolic Biophysics and Applied Pharmacology (LR12ES02), Department of Biophysics, Faculty of Medicine Ibn El Jazzar of Sousse, University of Sousse, Sousse 4002, Tunisia; nadia_jaidane@famso.u-sousse.tn (N.J.); wejdene.mansour@gmail.com (W.M.); tilouchelamia@gmail.com (L.T.); 2 Higher School of Health Sciences and Techniques of Monastir, University of Monastir, Monastir 5000, Tunisia; 3 Team ‘Resist’, UMR1184 ‘Infectious Disease Models and Innovative Therapies (IDMIT)’ (INSERM, University Paris-Saclay, CEA), Faculty of Medicine, University Paris-Saclay, 94270 Le Kremlin-Bicêtre, France; souadfayad6@gmail.com (S.F.); dr.allaham@hotmail.com (N.A.A.L.); 4 French National Reference Center for Antibiotic Resistance: Carbapenemase-Producing Enterobacterales, 94270 Le Kremlin-Bicêtre, France; 5 Department of Bacteriology-Hygiene, Bicêtre Hospital, APHP Paris-Saclay, 94270 Le Kremlin-Bicêtre, France; 6 Unité Antibiorésistance et Virulence Bactériennes, ANSES—Université de Lyon, 69007 Lyon, France; pierre.chatre@anses.fr (P.C.); pauline.francois@anses.fr (P.F.); laetitia.dufraysseix@anses.fr (L.D.F.); marisa.haenni@anses.fr (M.H.); 7 Laboratory of Bacteriology, Military Hospital of Tunis, Tunis 1008, Tunisia; aymanbouaziz02@gmail.com (A.B.); farouk.barguellil@gmail.com (F.B.); 8 Laboratory of Microorganisms and Environment, Molecular Diagnostic Tools and Emerging and Re-Emerging Infections (LR19DN03), Military Hospital of Tunis, Tunis 1008, Tunisia; 9 Institut de Chimie des Substances Naturelles, CNRS, Université Paris-Saclay, 91190 Gif-sur-Yvette, France; bogdan.iorga@cnrs.fr; 10 Laboratory Medicine Department, Faculty of Applied Medical Sciences, Al Azhar University, Gaza P.O. Box 1277, Palestine

**Keywords:** *Escherichia coli*, *mcr-1.1*, colistin resistance, plasmid-mediated resistance, Tunisia, IncI2, IncX4, CirA, Fiu, TonB, cefiderocol resistance

## Abstract

**Background/Objectives**: The emergence of plasmid-mediated *mcr* genes has enabled horizontal dissemination of resistance to colistin, a last-resort antibiotic against multidrug-resistant Enterobacterales. In Tunisia, genomic data on *mcr*-positive *Escherichia coli* are still limited. This study reports the genomic characterization of human clinical *mcr*-positive *E. coli* isolates from the Military Hospital of Tunis. **Methods:** Between August 2023 and March 2025, seven *E. coli* isolates with low-level colistin-resistance (MIC = 4–8 µg/mL) were collected from six patients. They were characterized by antibiotic susceptibility testing and WGS to determine resistome, MLST, genetic relatedness, and plasmid content. **Results:** The *E. coli* isolates belonged to diverse sequence types (STs), except for two isolates collected from the same patient 2.5 months apart, which were highly related. Overall, this pattern is consistent with a polyclonal spread. The *mcr-1.1* gene was located on IncI2 (*n* = 5) or IncX4 (*n* = 2) plasmids, which exhibited high similarity both among themselves and in comparison with plasmids previously reported in human and livestock isolates. Most isolates were multidrug-resistant, harboring acquired resistance genes to multiple antibiotic classes, and chromosomal mutations conferring fluoroquinolone resistance. Three isolates additionally carried chromosomal insertions of the *bla*_CTX-M-55_ gene. Resistance to cefiderocol was observed in one isolate and was associated with CirA and Fiu truncation. **Conclusions:** These findings highlight ongoing dissemination of *mcr-1.1*-positive *E. coli* isolates in Tunisia, primarily driven by plasmid transfer. Continuous genomic surveillance and One Health-oriented antibiotic stewardship are essential to limit the spread of colistin-resistance and the emergence of resistance to newer agents such as cefiderocol.

## 1. Introduction

Colistin remains a last-resort antibiotic to treat human infections caused by multidrug-resistant (MDR) Gram-negative bacteria, particularly carbapenemase-producing *Enterobacterales* [1]. Since the discovery in China in 2015, the plasmid-mediated *mcr-1* gene, and to a lesser extent a few of its *mcr-2* to *mcr-10* variants, have been reported from humans, animal, and environmental sources, making it a One Health issue [2,3]. Nevertheless, while the proportions of *mcr*-positive *E. coli* in livestock animals reached alarming levels worldwide, the occurrence of these isolates in human clinical isolates remained limited [4,5].

In Tunisia, colistin has been used in veterinary medicine for decades, leading to the emergence and dissemination of *mcr-1*-positive *E. coli* in several livestock sectors [6,7,8]. In most cases, the *mcr-1* gene was found co-localized with the *bla*_CTX-M-1_ gene on IncHI2 plasmids, even though IncP carriage was also reported. In Tunisian hospitals, colistin remains a last-resort therapeutic option for the treatment of infections caused by carbapenem-resistant bacteria. In clinical settings, *mcr-1*-positive *E. coli* was first reported in Tunisia in 2019 through a multicentric study describing four isolates associated with urinary tract infections [9]. However, the first detailed characterization of an *mcr*-carrying plasmid in Tunisian hospitals only dates back to 2023, when an *mcr-1*-harboring *E. coli* isolate recovered from a prosthetic joint infection at Sahloul Hospital in Sousse was described [10]. The isolate belonged to ST224 and carried the *mcr-1.1* gene on an IncI2 plasmid lacking the typical IS*Apl1* flanking elements, suggesting increased stability of the resistance cassette [10].

Given the importance of colistin in human medicine, especially in countries without access to novel antibiotics such as cefiderocol or novel ß-lactam/inhibitor combinations, clinical microbiology laboratories are monitoring the emergence of *mcr*-positive Enterobacterales. Cefiderocol is a novel siderophore cephalosporin that exploits bacterial iron transport systems to enter the periplasmic space, thereby overcoming several resistance mechanisms, including reduced outer membrane permeability and efflux pumps [11]. Owing to its stability against most clinically relevant β-lactamases, including carbapenemases, cefiderocol has become an important last-line therapeutic option for severe infections caused by multidrug-resistant Gram-negative bacteria [12]. However, reports of reduced susceptibility and resistance to cefiderocol have increasingly emerged worldwide, even among isolates without prior exposure to the drug, raising concerns regarding the preservation of its clinical efficacy [13]. Therefore, continuous surveillance of cefiderocol susceptibility, particularly among strains harboring transferable resistance genes such as *mcr-1*, is becoming increasingly important.

Here, a retrospective study conducted at the Military Hospital of Tunis between 2023 and 2025 revealed seven *mcr*-positive *E. coli* isolates recovered from six patients. WGS was used to characterize the *mcr-1*-positive *E. coli* isolates, determine the genetic context of the *mcr* genes, and identify potential routes of dissemination within the hospital.

## 2. Results

### 2.1. Clinical Characteristics

**Figure 1:**
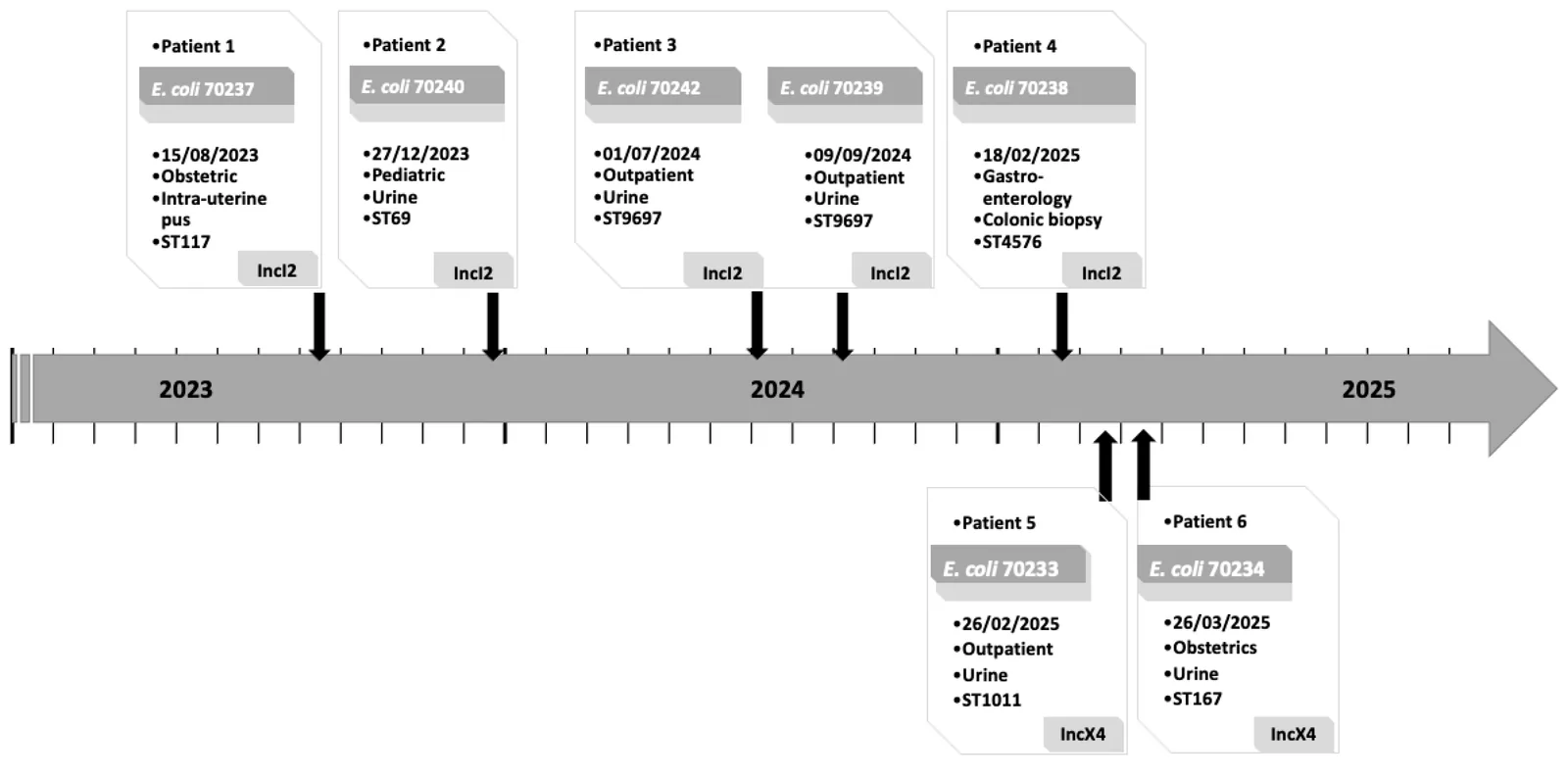
Timeline of *mcr-1*-positive *E. coli* isolates recovered from six patients in a military hospital in Tunisia (August 2023–March 2025). Dates of isolation, patient identifiers, isolate numbers, STs, and hospital wards are indicated. The plasmid carrying the *mcr-1* gene (IncI2 or IncX4) is indicated for each isolate.

Between August 2023 and March 2025, a total of 3104 *E. coli* isolates were recovered from clinical specimens processed at the Laboratory of Bacteriology of the Military Hospital of Tunis, Tunisia. Among these, seven isolates (7/3104; 0.2%) that were isolated from six patients exhibited low-level resistance to colistin (MIC = 4–8 μg/mL) (Figure 1).

As two isolates were collected from the same patient 2.5 months apart, both were retained for characterization. Colistin-resistant isolates originated from diverse clinical samples, including urine, intra-abdominal pus, tissue biopsy, and blood cultures (Table 1). Patients were from different wards, namely pediatrics (*n* = 1), gynecology/obstetrics (*n* = 2), gastroenterology (*n* = 1), and outpatient settings (*n* = 2). Two patients originated from urban areas and four from rural regions. Five out of the six patients reported having contacts with livestock animals and none reported a recent trip abroad. Clinical review indicated that the recovered isolates were associated with clinically relevant infections rather than incidental colonization. Urinary isolates originated from patients diagnosed with symptomatic urinary tract infections or presenting with significant urological disorders, including bladder cancer requiring surgical management. Most patients had underlying comorbidities such as diabetes mellitus, chronic kidney disease, hypertension, hypothyroidism, inflammatory bowel disease, or malignancy, and several had recent healthcare exposure or previous hospitalization. Appropriate antimicrobial therapy was initiated according to the clinical presentation, including cefixime, amoxicillin-clavulanate, piperacillin-tazobactam combined with metronidazole, or cefotaxime-based regimens, while one patient required surgical intervention. Clinical outcomes were favorable in all evaluable patients, underscoring the clinical relevance of the recovered isolates. (Table S1).

### 2.2. Antimicrobial Susceptibility Patterns

Susceptibility testing by BMD using a large panel of 47 antibiotics showed that six out of the seven isolates exhibited an MDR phenotype (Table 2) [14]. Isolates remained susceptible to piperacillin/tazobactam, all carbapenems, aminoglycosides (amikacin, gentamicin, tobramycin), fosfomycin, tigecycline, eravacycline and to the new β-lactam/β-lactamase inhibitor combinations, including meropenem–vaborbactam, ceftazidime–avibactam, and aztreonam–avibactam. Three isolates were resistant to ceftolozane/tazobactam, while resistance to cefiderocol (MIC = 16 mg/L) was observed in one isolate (# 70237), and reduced susceptibility (MICs = 0.5–2 mg/L) was observed in three isolates (# 70239, 70242 and 70240) (Table 2).

### 2.3. Molecular Characterization of Isolates

*E coli* isolates belonged to six different STs (ST69, ST1011, ST117, ST167, ST4576, and ST9697 (*n* = 2) and to distinct phylogroups and serogroups, including phylogroup D (serogroups O160:H16, O51:H42, and O17/O44:H18), phylogroup A (O89:H21), phylogroup G (O45:H4), and phylogroup B1 (O8:H10), reflecting antigenic diversity (Table 1).

Analysis of the resistome confirmed the presence of the *mcr-1.1* allele in all isolates (Table 1). In addition, resistance to several other antibiotic families was observed, such as β-lactams (*bla_TEM-1B_* (n = 4), *bla_CTX-M-55_ (n* = 2)), tetracyclines (*tet(A)*), trimethoprim (*dfrA12*, *dfrA14*, *dfrA17*), sulfonamides (*sul1*, *sul2*) and chloramphenicol (*floR*) (Table 1). Six out of seven isolates also carried amino acid changes in *GyrA* (four with the combination S83L/D87N, and one with S83L alone) and *ParC* (S80I) enzymes, conferring resistance to fluoroquinolones. In addition, one isolate (70234) carried the plasmid-mediated *qnrS1* gene. In contrast, *E. coli* isolate 70238, characterized by a rare ST4576, displayed the *mcr-1* gene but no other antimicrobial resistance gene or point mutation associated with resistance, in accordance with the observed phenotype (Table 1).

The two isolates recovered from the same patient (70239 and 70242) belonged to the same ST, differed only by 17 SNPs (based on core-genome SNP analysis), and displayed the same resistome and antimicrobial susceptibility profile, suggesting the persistence in the host of a single clone.

### 2.4. Cefiderocol Resistance

Three of the seven isolates displayed cefiderocol MICs of ≤0.03 mg/L, consistent with the low MICs typically reported for wild-type *E. coli* strains (approximately 0.06–0.12 mg/L) [15]. In contrast, four isolates exhibited elevated cefiderocol MICs compared with those expected for wild-type *E. coli*. Although three of these isolates remained within the clinical susceptibility breakpoint, one was classified as resistant.

Because cefiderocol enters Gram-negative bacteria via TonB-dependent iron transport systems, alterations affecting TonB or TonB-dependent siderophore receptors including CirA and Fiu in Enterobacterales can impair antibiotic uptake and reduce susceptibility [16,17]. Additional resistance mechanisms include alterations in penicillin-binding protein 3 (PBP3), which decrease cefiderocol affinity. When combined with β-lactamases exhibiting even weak cefiderocol hydrolytic activity, these alterations may act synergistically to confer clinically relevant reductions in susceptibility or frank resistance [16]. To investigate the molecular basis of these elevated MICs, the *ftsI*, *tonB*, *Fiu* and *cirA* genes, encoding, respectively the PBP3, TonB energy transducer, and the two catecholate siderophore receptors Fiu and CirA, were analyzed.

**Figure 2:**
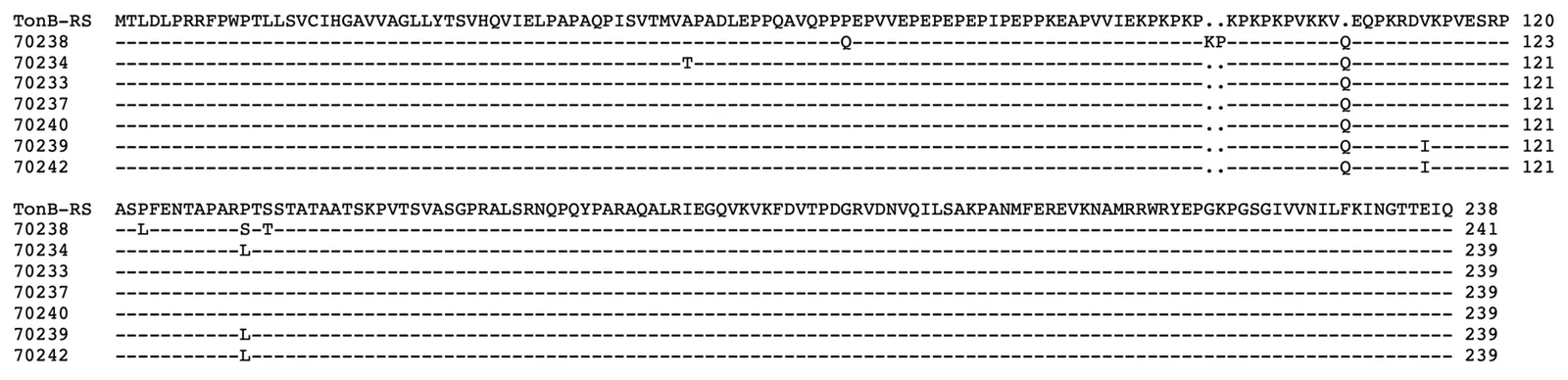
Amino acid sequence alignment TonB from *mcr-1* producing *Escherichia coli* isolates from this study with that of the *Escherichia coli* ATCC 25922 strain (RS: RefSeq; GenBank accession no. CP009072). Identical amino acids are indicated by dashes, and missing ones by dots.

No mutation was found in the *ftsl* gene, as compared to that of *E. coli* MG1665 (RefSeq: GCF_056500995.1). Similarly, only a limited number of amino acid changes were identified in TonB compared with the reference sequence of *E. coli* ATCC 25922 (CP009072) (Figure 2). In isolate 70238, which remained fully susceptible to cefiderocol, a two-amino-acid insertion (KP) after residue 96 was observed. The preservation of cefiderocol susceptibility in this isolate suggests that this insertion is unlikely to have a major impact on antibiotic uptake or susceptibility.

**Figure 3:**
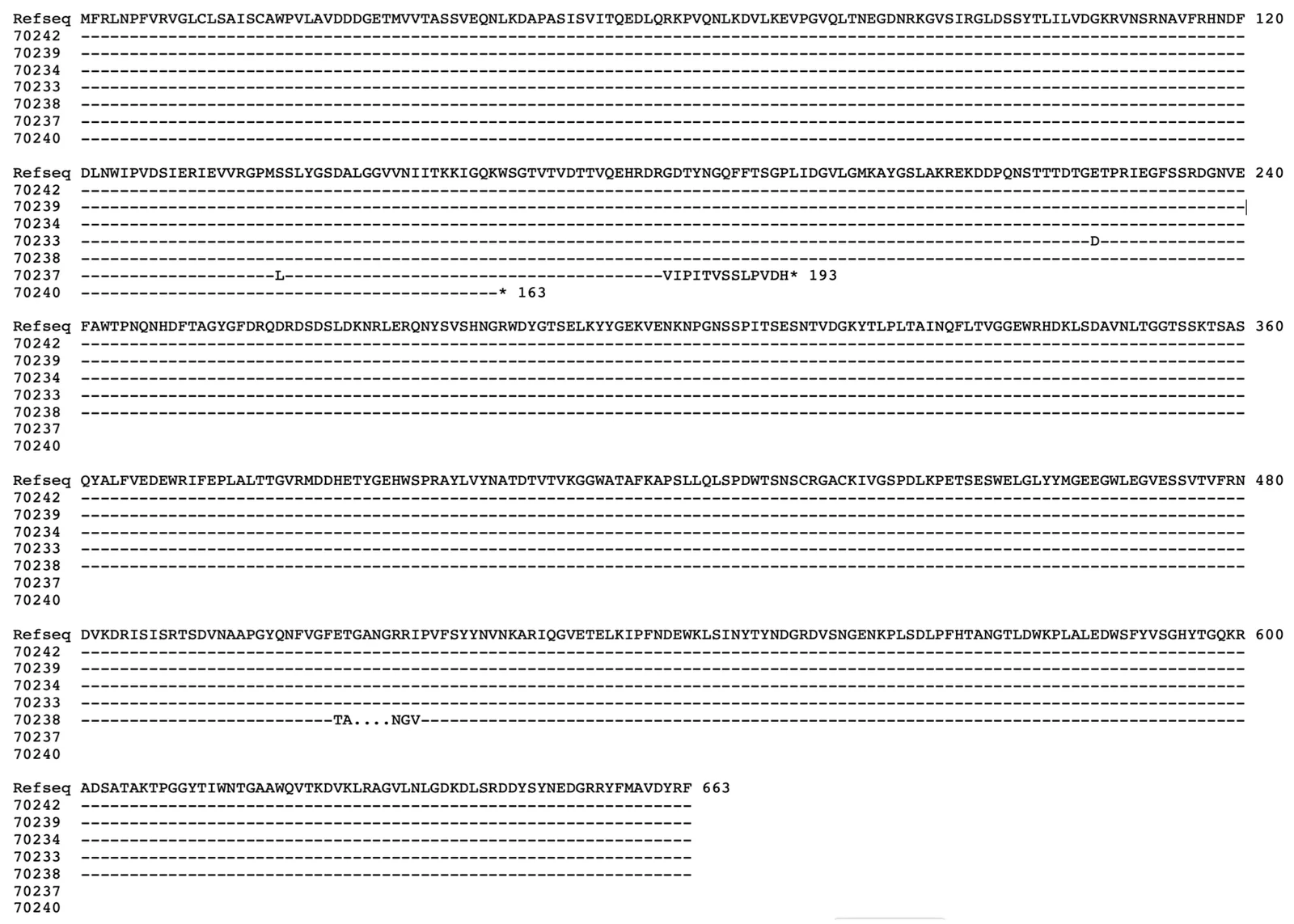
Amino acid sequence alignment of catecholate siderophore receptor CirA protein from *mcr-1*-producing *Escherichia coli* isolates from this study with that of the *Escherichia coli* ATCC 25922 strain (RefSeq; GenBank accession no. CP009072). Identical amino acids are indicated by dashes, gaps by dots and premature interruptions by asterisks (*).

Compared to strain *E. coli* ATCC 25922, *cirA* nucleotide alterations resulting in premature stop codons were identified in one cefiderocol-resistant isolate (70237; MIC, 16 mg/L) and one isolate with reduced susceptibility (70240; MIC, 0.5 mg/L) (Figure S1, Table 2). These mutations resulted in truncated proteins of 163 and 193 amino acids (aa) respectively (Figure 2), and may contribute to the increased cefiderocol MICs observed in these isolates. In the isolates 70239 and 70242 (MIC = 0.5 and 2 mg/L, respectively), no mutation in *cirA* were found. In isolate 70238, a deletion of four aa at the C-terminus of CirA was also observed, however this isolate was fully susceptible to cefiderocol; suggesting that this deletion is unlikely to contribute to cefiderocol resistance (Figure 3).

**Figure 4:**
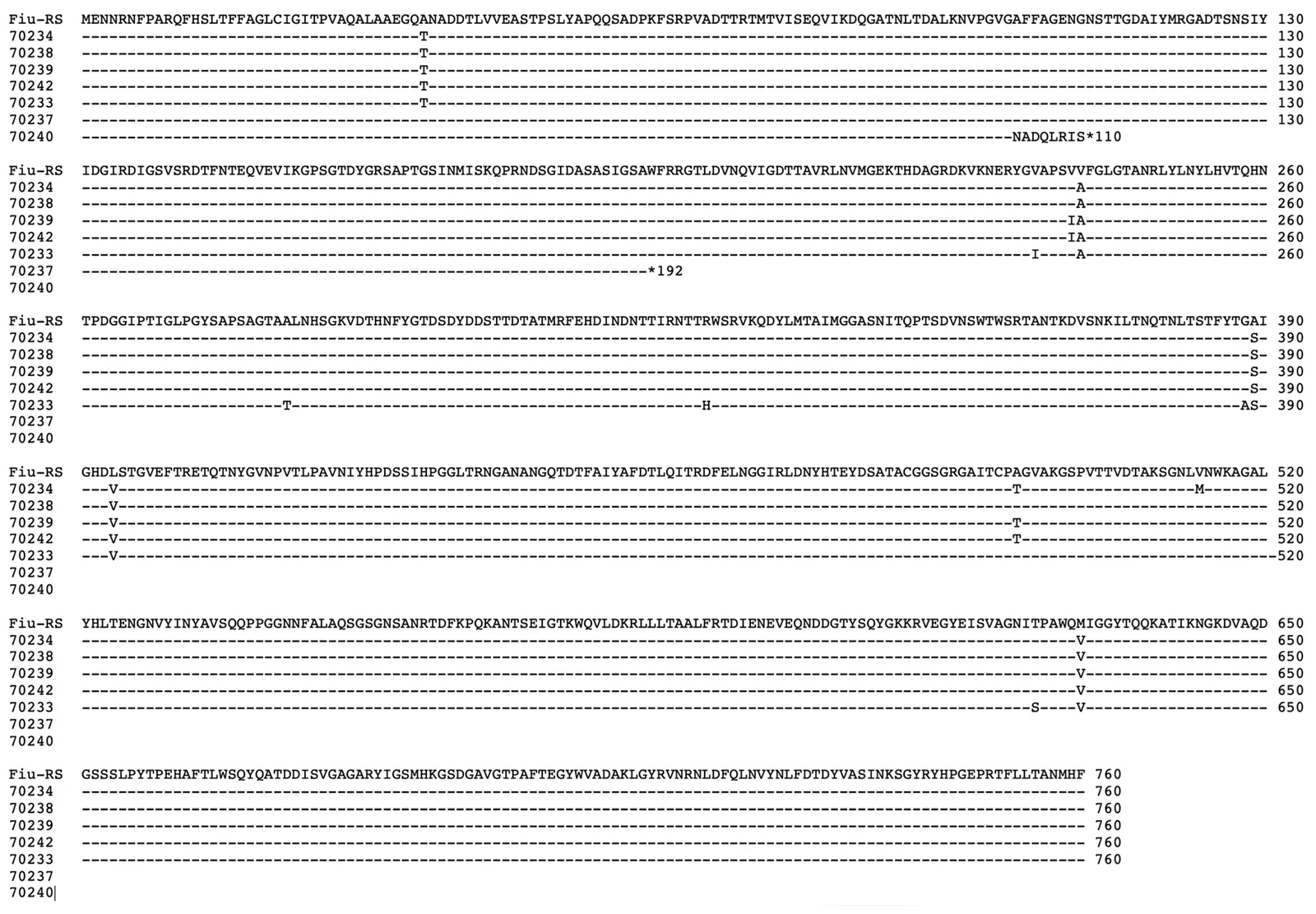
Amino acid sequence alignment of Fiu protein, a TonB-dependent outer membrane receptor for catecholate-type siderophore uptake from *mcr-1*-producing *Escherichia coli* isolates from this study with that of the *Escherichia coli* ATCC 25922 strain (RS: RefSeq; GenBank accession no. CP009072). Identical amino acids are indicated by dashes, missing ones by dots and premature interruptions by *.

Compared with *E. coli* ATCC 25922, *fiu* nucleotide alterations predicted several amino acid substitutions as well as premature stop codons in the cefiderocol-resistant isolate 70237 (MIC, 16 mg/L) and in isolate 70240 (MIC, 0.5 mg/L), both of which also harbored *cirA*-inactivating mutations (Figure S2, Table 2). These premature stop codons resulted in truncated Fiu proteins of 110 and 192 amino acids, respectively (Figure 4), likely contributing to the increased cefiderocol MICs by impairing antibiotic uptake. In contrast, isolates 70239 and 70242 (MICs, 0.5 and 2 mg/L, respectively) harbored only amino acid substitutions in Fiu. Because these substitutions were also identified in fully cefiderocol-susceptible isolates, they are unlikely to be sufficient, on their own, to explain the reduced cefiderocol susceptibility observed in these isolates (Figure 4).

### 2.5. Genetic Support of mcr-1.1 and bla_CTX-M-55_ Genes

Among the seven *E. coli* isolates, five carried the *mcr-1.1* genes on IncI2 plasmids (including three isolates co-harboring the *bla*_CTX-M-55_ gene) and two on IncX4 plasmids (Figure 1). IncI2 plasmids were identified in the five oldest isolates (August 2023 to March 2025), while IncX4 were identified in the two most recent ones (February–March 2025).

**Figure 5:**
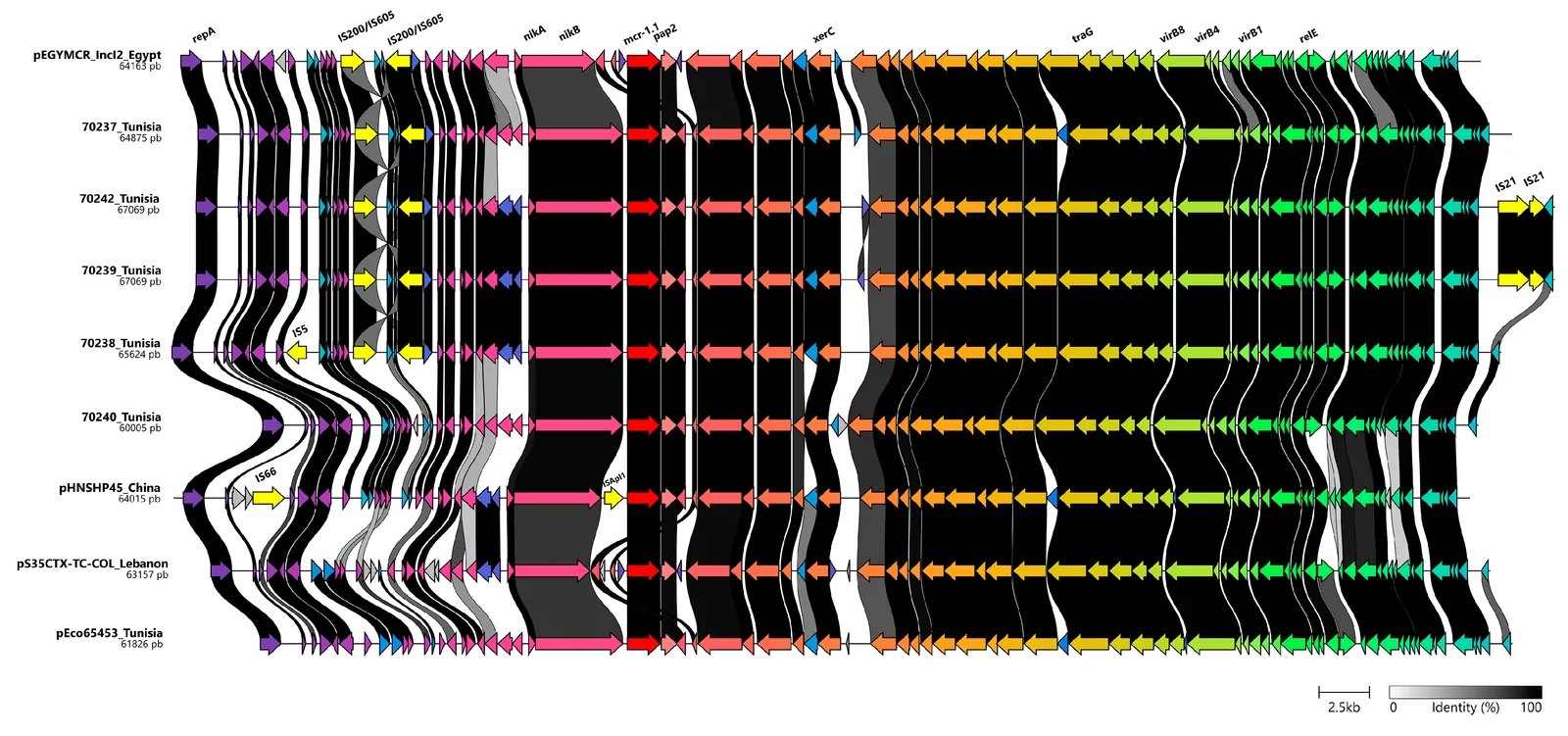
Comparison of *mcr-1*-carrying plasmids belonging to the IncI2 type. The name and size of each plasmid are indicated in front of each sequence. The nucleotide sequence identity between each coding region is represented with a color gradient ranging from white (0% identity) to black (100% identity). A few genes of interest are also specified: *repA*, *TraG*, *virB4*, *relE*, *mcr-1*, *pap2.* IncI2 plasmids corresponded to pHNSHP45 (GenBank: KP347127) [3], plasmid pECO-TUN-65453 (GenBank: PRJNA1238567) [10], previously reported in humans from Tunisia plasmid pEGYMCR_IncI2 (GenBank: MT499885.1) from Egypt [18] and plasmid pS35CTX-TC-COL (GenBank: OZ026767.1) from Lebanon [19].

The five IncI2 plasmids were highly similar to each other (95 to 100% coverage and 99 to 100% identity) and were also closely related to the original plasmid reported by Liu et al. (pHNSHP45, [KP347127] [3]) and those previously reported in humans from Tunisia (pECO-TUN-65453 [PRJNA1238567] [10]) and from broilers in Egypt (pEGYMCR_IncI2 [MT499885.1] [18]) and Lebanon (pS35CTX-TC-COL [OZ026767.1] [19]) (Figure 5). The IncI2 plasmid identified in isolate 70240 shared higher homologies with the pECO-TUN-65453 plasmid isolated in 2023 at the Sahloul hospital (Sousse, Tunisia) than with the three other IncI2 plasmids from this study, which shared more similarities with a plasmid from Egypt, notably due to the presence of a 3.9 kbp element bracketed by IS*200*/IS*605* family transposases [18].

**Figure 6:**
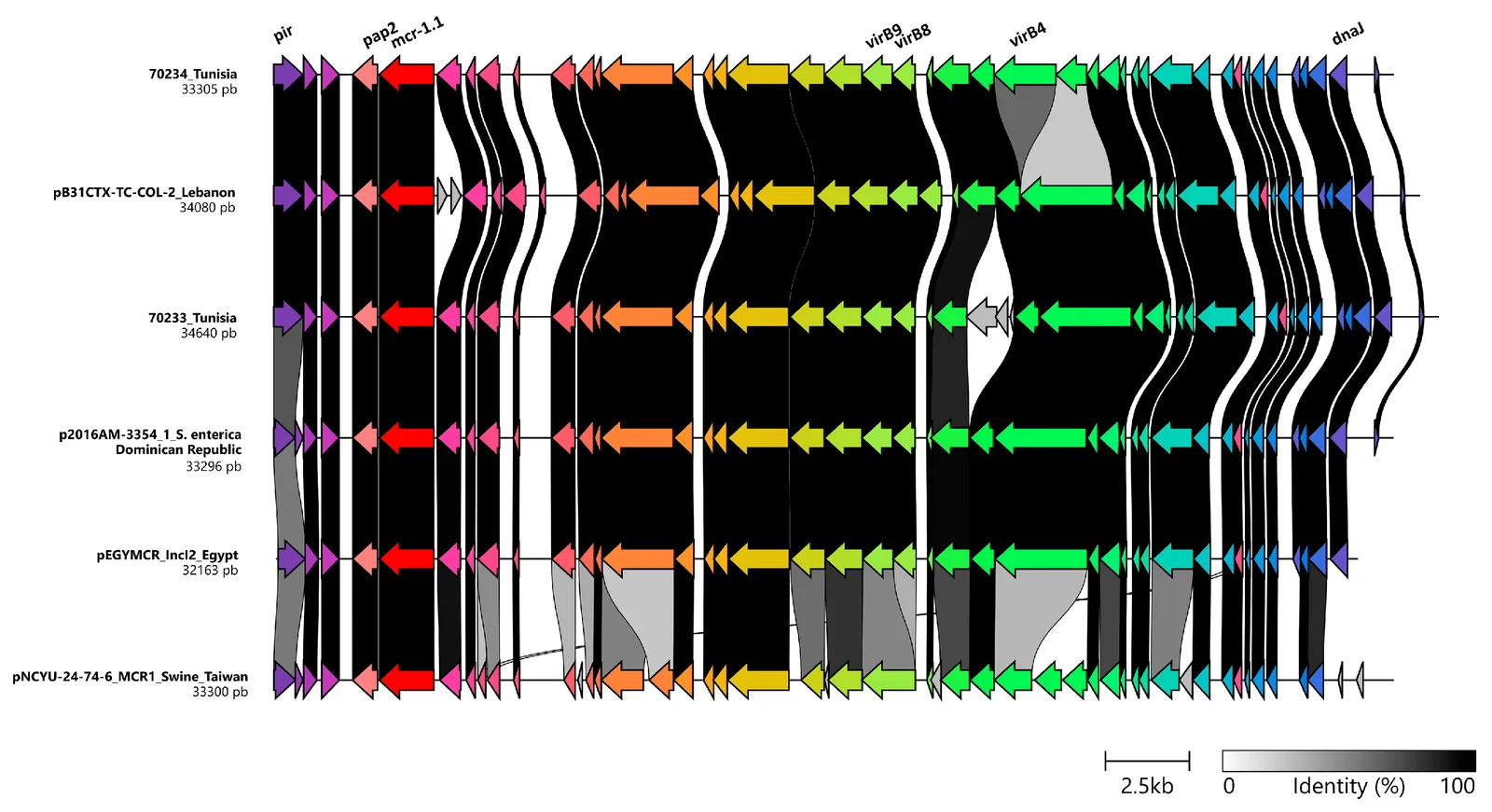
Comparison of *mcr-1*-containing plasmids belonging to the IncX4 type. The name and size of each plasmid are indicated in front of each sequence. The nucleotide sequence identity between each coding region is represented with a color gradient ranging from white (0% identity) to black (100% identity). A few genes of interest are also specified: *repA*, *TraG*, *virB4*, *relE*, *mcr-1*, *pap2.* IncX4 plasmids corresponded to p2016AM-3354 (GenBank: CP092291) from a *Salmonella enterica* isolate [20], pEGYMCR_IncX4 (GenBank: MT499886) from Egypt [18], pB31CTX-TC-COL-2 (GenBank: OZ026879) from Lebanon [19] and pNCYU-24-74-6_MCR1 (GenBank: CP042644.1) from swine in Taiwan [21].

Likewise, the IncX4 plasmids identified here shared high similarities with reported from broilers in Egypt (pEGYMCR_IncI2 [MT499886] [18]) and Lebanon (pB31CTX-TC-COL-2 [OZ026879] [19]) (Figure 6), but also with plasmids from a *Salmonella enterica* isolate (p2016AM-3354_1 [CP092291.1] [20]) collected from a US traveler and from an *E. coli* (pNCYU-24-74-6_MCR1 [CP042644.1] isolate collected in swine in Taiwan [21]. Only the Lebanese isolate [OZ026879] harbored an IS element upstream of the *mcr-1.1* gene.

Isolates 70237, 70239 and 70242, belonging to two different STs (ST117 and ST9697) and harboring an IncI2 plasmid-borne *mcr-1.1* gene, additionally carried the ESBL *bla*_CTX-M-55_ gene. In these three *E. coli* isolates, the *bla*_CTX-M-55_ gene was located on a mobile element made of an IS*Ecp1* element, located 45 bp upstream of the *bla*_CTX-M-55_ gene, and the *wbuC* gene located 46 bp downstream, which was inserted at the same locus on the chromosome of both strains. However, in the *E. coli* 70237 ST117 isolate, IS*Ecp1* was truncated (∆IS*Ecp1*).

## 3. Discussion

Given the low number of *mcr-1*-positive *E. coli* isolates reported in human medicine, the detection of six patients with such isolates in a single Tunisian tertiary care center over three years represents an epidemiological signal whose significance needs to be assessed.

Two isolates (70239 and 70242) were recovered from the same patient during outpatient visits 2.5 months apart and were analyzed to assess genetic relatedness and explore potential resistance selection over time. The isolates differed by only 17 SNPs, confirming close genetic relatedness, and exhibited identical antimicrobial susceptibility profiles, suggesting that these SNPs had no impact on resistance phenotypes. Although it is possible that these SNPs accumulated during the 2.5-month colonization period, this appears unlikely. Given an estimated evolutionary rate of approximately 6–7 SNPs per year in the *E. coli* genome, it is more plausible that the patient had been colonized by the *mcr-1.1*-positive strain for a longer period or that colonization involved an already genetically diverse bacterial population.

Five out of the six patients reported having contact with livestock animals, supporting an extra-hospital source of contamination, possibly from a non-urban environment. This hypothesis is further supported by the identification of six different STs suggesting community acquisition and importation into the hospital. Four out of the six identified STs (167, 1011, 117, 69) have recognized zoonotic or global epidemiological importance and have been recurrently associated with *mcr* carriage. ST167 is a globally distributed clone, which seems prone to interspecies transmission [22]. In France, it has recently been described as the main *mcr-1*-carrying *E. coli* lineage in the bovine sector [22,23]. ST1011 is a zoonotic lineage originally associated with environmental and poultry sources, but rarely with humans [22,23,24,25,26]. In Lebanon, *mcr-1*-positive *E. coli* ST1011 was detected in poultry, a patient, and a farm worker, suggesting cross-species transmission [27]. ST117 also has avian origins even though it has now spread to multiple hosts including livestock and humans [28]. It often harbours resistance genes such as *bla*_TEM_, *cat1*, *tetB* and *sul1*/*sul2*; similar profiles occurred in poultry isolates from Poland, Egypt, and Switzerland [28]. In Tunisia, *E. coli* ST117 carrying *mcr*-1 and *bla*_CMY-2_ have been found in healthy chickens, confirming poultry as a reservoir [29]. ST69 is globally distributed and considered as a pandemic high-risk clone, which has only been reported in Tunisia from two wildlife isolates carrying *bla*_CTX-M-15_ and *qnrS1* [30].

Although the presence of *mcr-1.1* did not influence the immediate therapeutic management of the patients included in this study, since all isolates remained susceptible to several clinically relevant antimicrobial agents, its detection remains epidemiologically important. The principal concern is the dissemination of plasmid-mediated colistin resistance, which can readily spread between bacterial populations and jeopardize one of the last-resort therapeutic options for multidrug-resistant Gram-negative infections. In Tunisia, only isolated reports of clinical *mcr-1*-positive *E. coli* have previously been described from another geographical region [10]. Therefore, our findings suggest that plasmid-mediated colistin resistance is no longer geographically restricted and may be spreading at the country level. Given the widespread use of colistin in Tunisia, these findings should be considered as an early warning supporting the implementation of continuous surveillance and infection control measures to limit further dissemination.

Alterations or inactivation of the CirA and/or Fiu iron transporter have previously been associated with cefiderocol resistance in clinical isolates [15,31,32]. In the present study, only one isolate exhibited cefiderocol resistance according to EUCAST criteria. Interestingly, truncating *cirA* and *fiu* mutations were identified in both the resistant isolate (70237; MIC = 16 mg/L) and an isolate with reduced susceptibility to cefiderocol (70240; MIC = 0.5 mg/L), suggesting that *cirA* and/or *fiu* disruption alone is insufficient to confer a resistant phenotype. These findings suggest that additional resistance mechanisms, such as alterations in other iron uptake systems, β-lactamase activity, or as-yet unidentified mechanisms, contribute to the high cefiderocol MIC observed in isolate 70237 [17].

In two additional isolates, 70239 and 70242, no CirA alterations were identified, and only a few amino acid substitutions were detected in Fiu, despite elevated cefiderocol MICs of 0.5 and 2 mg/L, respectively. These Fiu substitutions are unlikely to account for the increased cefiderocol MICs, as they were also observed in fully susceptible isolates; however, their potential contribution cannot be excluded and warrants further investigation (Figure 4).

Although high-level cefiderocol resistance is generally thought to result from the accumulation of multiple mechanisms, including reduced uptake, β-lactamase-mediated hydrolysis, and/or decreased target susceptibility (for example through PBP3 alterations), the role of individual β-lactamases remains incompletely defined [16,33]. In contrast to β-lactamases such as NDM, certain PER, VEB, and GES variants, or hyperproduced AmpC enzymes, CTX-M-55 has not been established as a major cefiderocol resistance determinant. Nevertheless, CTX-M-55 may have contributed to the reduced cefiderocol susceptibility observed in these isolates. This enzyme displays enhanced catalytic efficiency (kcat/Km) against expanded-spectrum cephalosporins compared with CTX-M-15 [34], and its contribution may be amplified in the context of impaired siderophore-mediated uptake. This could partly account for the higher cefiderocol MIC observed in *E. coli* 70237 (MIC, 16 mg/L) compared with *E. coli* 70240 (MIC, 0.5 mg/L), as *E. coli* 70237 carried both truncated cirA/fiu genes and the *bla*_CTX-M-55_ gene. Further functional studies are required to confirm this hypothesis.

Alterations or inactivation of the *cirA* gene have previously been reported to arise during cefiderocol therapy [15,31,32]. In some cases, these mutations have been described even in the absence of cefiderocol selection pressure and despite their potential fitness cost [34]. In addition, cefiderocol resistance has increasingly been reported outside clinical settings, with resistant strains detected in aquatic environments such as surface waters in Ghana and the Baltic Sea [35,36].

While clonal transmission could be ruled out in our collection of isolates, dissemination of the plasmids most likely occurred, either inside or outside the hospital. Indeed, five strains carried the *mcr-1.1* gene on highly similar IncI2 plasmids, and two on IncX4 plasmids, suggesting the spread of two distinct plasmid backbones carrying *mcr-1* colistin resistance determinants. This finding extends our previous observation from Sahloul hospital [10], where the *mcr-1.1* gene was located on an IncI2 plasmid. Here, a highly related IncI2 plasmid was identified in four isolates, while the fifth one was slightly different, suggesting persistence and/or reintroduction of plasmid lineages within Tunisian healthcare environments.

Both IncI2 and IncX4 plasmids have been widely reported to be highly transferable across bacterial species [2,4]. Globally, the IncI2 plasmid type was the earliest and remains the most frequently encountered vehicle, followed by IncX4 and IncHI2, together accounting for over 90% of reported *mcr-1*-bearing plasmids [37,38]. Despite their widespread distribution, the reasons for their dominance and their effects on host fitness remain poorly understood. The usually high transfer capacity of both IncX4 and IncI2 may probably explain their wide geographical dissemination and their occurrence in humans and in a large variety of animal hosts [27]. In Tunisia, *mcr-1*-positive *E. coli* have been described in animal, environmental, and human sources [9,39], but detailed plasmid characterizations were scarce until the report of an isolate from the Sahloul hospital [10].

Additionally, three *E. coli* isolates (belonging to ST9697 and ST117) carried the *bla*_CTX-M-55_ gene, which remains scarce in Tunisian *E. coli* isolates of human and animal origin [7,40]. The gene was chromosomally embedded through the integration of an IS*Ecp1*-*bla*_CTX-M-55_-*wbuC* (orf477) element at the same genetic locus. This configuration mirrors previous findings in Tunisia, where two studies reported similar genetic environments among CTX-M-ESBL-producing *E. coli* from chicken faeces, retail meat, and urban and industrial wastewater, indicating a conserved chromosomal integration pattern of this resistance determinant [7,40]. The presence of IS*Ecp1* upstream of *bla*_CTX-M-55_ suggests its role in mobilization and high-level expression of *bla_CTX-M_* alleles [41]. The truncated IS*Ecp1* (∆IS*Ecp1*) found in the ST117 strain likely results from recombination or partial deletion events mediated by other insertion sequences. The conservation of *orf477* downstream of *bla*_CTX-M-55_ supports the existence of a common ancestral module involved in chromosomal capture of this gene. Collectively, the chromosomal localization of *bla*_CTX-M-55_ in similar genetic contexts across distinct sequence types highlights its potential for stable vertical transmission, complementing the global plasmid-mediated spread of this resistance determinant.

This study has several limitations. First, the number of positive isolates was small, and all isolates were collected from a single tertiary-care hospital, which may limit the generalizability of our findings to other healthcare settings or geographic regions. Second, isolate selection was based on phenotypic colistin resistance rather than systematic molecular screening of all *E. coli* isolates, potentially underestimating the prevalence of *mcr*-harboring isolates. Finally, contemporaneous animal, food, or environmental isolates were not available for comparison, precluding direct investigation of potential reservoirs and transmission pathways within a One Health framework. Nevertheless, the complete genomic characterization of all isolates and their associated plasmids, together with the detailed clinical data presented, provides valuable insights into the emergence and dissemination of plasmid-mediated *mcr-1.1* among clinical *E. coli* isolates in Tunisia.

## 4. Materials and Methods

### 4.1. Bacterial Isolates and Identification

Between August 2023 and March 2025, *E. coli* isolates with reduced susceptibility to colistin, as revealed by an automated VITEK 2 system (AST357 card; bioMérieux, Marcy l’Etoile, France), recovered from clinical specimens processed at the Laboratory of Bacteriology of Military Hospital of Tunis, Tunisia, were included. Inclusion criteria were one isolate per patient per month. MICs were interpreted according to EUCAST 2026 guidelines (https://www.eucast.org/; accessed on 30 June 2026). Colistin resistance was subsequently confirmed using the broth microdilution reference method, in accordance with EUCAST guidelines (https://www.eucast.org/eucastguidancedocuments, accessed on 30 June 2026). Identification of colistin-resistant *E. coli* isolates was confirmed using MALDI-TOF Biotyper (Bruker, Hamburg, Germany).

### 4.2. Antimicrobial Susceptibility Testing

Susceptibility profiles of *mcr-1*-positive bacteria were determined by broth microdilution (BMD) (Seq2Diag plates 1, 2, 3, Sensititre, ThermoFisher, Les Ulis, France) for 47 antibiotics, including novel beta-lactam and non-beta-lactam antibiotics of clinical and veterinary interest and interpreted according to EUCAST 2026 breakpoints (https://www.eucast.org/), except for veterinary-restricted agents interpreted using veterinary breakpoints set by CA-SFM vétérinaire 2023 criteria (https://www.sfm-microbiologie.org/2023/06/15/casfm-veterinaire-2023/; accessed on 30 June 2026). Tigecycline MICs were interpreted using EUCAST 2026 epidemiological cut-off values, ECOFFs, cefiderocol MICs were determined by BMD using the UMIC Cefiderocol^®^ kit (Biocentric, Bandol, France) under iron-depleted conditions and interpreted according to EUCAST 2026 guidelines.

### 4.3. Molecular Characterization

PCR screening for the *mcr-1* to *mcr-9* genes was performed [42,43]. Genomic DNA of the *mcr*-positive isolates was extracted using the NucleoSpin Microbial DNA extraction kit (Macherey-Nagel, Hoerdt, France) and libraries were prepared using the ligation sequencing kit (SQK-LSK114). Sequencing was performed on an Mk1B MinION sequencer using a R10.4.1 flow cell (FLO-MIN114, Nanopore, Oxford, UK), and basecalling was performed with Dorado v0.4.2. Mean sequencing depth ranged from 46x to 120x, and all genomes (chromosomes and plasmids) were fully circularized.

Reads were assembled using a previously described pipeline [44]. SQUAT was used to assess the quality of the assemblies and the contig circularization was assessed from the Autocycler output. Sequence types (STs) were obtained using MLST version 2.23.0 (https://github.com/tseemann/mlst; accessed on 30 June 2026) and gene annotation was performed with PROKKA version 1.14.5 [45,46]. Antimicrobial resistance motifs and plasmid incompatibility groups were determined using Abricate version 1.0.1 (https://github.com/tseemann/abricate; accessed on 30 June 2026) with the Resfinder v4.7.2, NCBI AMRFinder 4.2.7, CARD v4.0.1 and PlasmidFinder v2.0.1 databases. Virulence gene detection was performed using the Center for Genomic Epidemiology tools (https://www.genomicepidemiology.org/; VirulenceFinder v2.0.3; accessed on 30 June 2026). Phylogenetic analyses based on core-genome single nucleotide polymorphisms (SNPs) were performed using CSI Phylogeny 1.4.

Plasmids were compared to similar plasmids retrieved from the NCBI database using BLAST analysis (BLAST+ 2.15.0; https://blast.ncbi.nlm.nih.gov/Blast.cgi; accessed on 30 June 2026) and visualized using clinker version 0.0.29-1 (https://cagecat.bioinformatics.nl/tools/clinker; accessed on 30 June 2026) [47].

### 4.4. Nucleotide Sequence Accession Number

This whole genome shotgun project was deposited at DDBJ/ENA/GenBank under the BioProject PRJNA1392031 (see Table 1, for individual accession numbers).

## 5. Conclusions

Our study documents the silent dissemination of plasmid-mediated colistin resistance in Tunisia, with the identification of seven clinical *E. coli* isolates carrying *mcr-1.1* at the Military Hospital of Tunis between 2023 and 2025. Our findings support the hypothesis that Tunisia has entered a phase of sustained plasmid circulation, rather than sporadic introductions of *mcr-1*. The lack of IS*Apl1* flanking *mcr-1.1* in both IncX4 and IncI2 plasmids likely contributes to plasmid stability and persistence. The implementation of rapid diagnostic tools, such as lateral flow immunoassays [48], could facilitate timely infection control measures and help prevent nosocomial transmission.

Globally, strengthening genomic surveillance within a One Health framework, together with systematic screening upon admission to healthcare settings, is essential to prevent the spread of colistin-resistant isolates in hospitals. Notably, despite the absence of prior exposure to cefiderocol, which has not yet been approved for clinical use in Tunisia, the identification of *E. coli* isolates resistant to both colistin and cefiderocol is particularly concerning and warrants further investigation to elucidate the selective pressures driving this emergence.

## Figures and Tables

**Table 1:** Epidemiological and WGS data of the seven *mcr-1*-positive *E. coli*.

Patient						Isolates			Nanopore Whole-Genome Sequencing Results					
#	Lifestyle	Ward ^b^	Date of Isolation (m/d/y) ^c^	Site of Isolation	Tx ^d^	#	ST ^e^-Phylogroup	Sero Group	Depth.^f^	Contigs		Chromosome/Inc Group	Resistance Genes	Accession Numbers
										#	Size (pb)			
1	Urban	G/O	08.15.23	Intra-uterine pus	CTX OFL MNZ then DOX GEN	70237	117-G	O45:H4	101×	1	5,046,734	Chromosome	*bla*_CTX-M-55_, *aph(3″)-Ib*, *aph(6)-Id*, *tet(A)*, *sul2*, *floR*, *gyrA* (S83L, D87N) and *parC* (S80I)	JBTFHH000000000
										2	102,713	IncFIB, IncFIC	*aadA5*, *dfrA17*, *qacE*	
										3	64,875	IncI2	*mcr-1.1*	
										4	7136	ColRNAI		
										5	4110	Col(pHAD28)_1		
										6	4074	ColRNAI		
2	Urban	P	12.27.23	Urine	None	70240	69-D	O17/O44:H18	46×	1	5,121,343	Chromosome	*bla*_TEM-1B_, *gyrA* (S83L) and *parC* (S80I)	JBTFHF000000000
										2	114,972	IncFIB, IncFIA, IncY	*tet(B)*, *dfrA14*	
										3	82,440	IncB/O/K/Z		
										4	60,006	IncI2	*mcr-1.1*	
										5	34,185	IncX1, IncX10		
										6	5167	Col156		
3 ^a^	Rural	O	09.09.24	Urine	None	70239	9697-B1	O8:H10	119×	1	5,071,810	Chromosome	*bla*_CTX-M-55_, *tet(A)*, *sul2*, *floR*, *aph(3″)-Ib*, *aph(6)-Id*, *gyrA* (S83L, D87N) and *parC* (S80I)	JBWXYB000000000
										2	146,305	IncFIB, IncFII	*bla*_TEM-1B_, *sul2, aph(3″)-Ib*, *aph(6)-Id*, *dfrA5*	
										3	67,069	IncI2	*mcr-1.1*	
										4	4593	ColRNAI_1		
										5	4110	Col(pHAD28)_1		
										6	3223	Col440II_1		
3 ^a^	Rural	O	07.01.24	Urine	None	70242 ^a^	9697-B1	O8:H10	72×	1	5,102,612	Chromosome	*bla*_CTX-M-55_, *tet(A)*, *sul2*, *floR*, *aph(3″)-Ib*, *aph(6)-Id*, *gyrA* (S83L, D87N) and *parC* (S80I)	JBTFHE000000000
										2	143,765	IncFIB, IncFII	*bla*_TEM-1B_, *sul2*, *aph(3″)-Ib*, *aph(6)-Id*, *dfrA5*	
										3	67,069	IncI2	*mcr-1.1*	
										4	4593	ColRNAI		
										5	4110	Col(pHAD28)_1		
										6	3223	Col440II		
4	Rural	G	02.18.25	Colonic biopsy	PTZ, MNZ	70238	4576-D	O51:H42	82×	1	5,383,636	Chromosome		JBTFHG000000000
										2	102,727			
										3	65,624	IncI2	*mcr-1*	
5	Rural	O	02.26.25	Urine	CFX	70233	1011-D	O160:H16	101×	1	5,011,099	Chromosome	*bla*_TEM-1B_, *gyrA* (S83L, D87N) and *parC* (S80I)	JBTFHJ000000000
										2	137,528	IncFIC, IncFIB	*tet(A)*, *sul1*, *drfA12, mph(A)*, *aadA2*, *qacE*	
										3	114,635	IncFIB		
										4	108,576	IncI1-I	*floR*	
										5	94,967	p0111		
										6	34,640	IncX4	*mcr-1.1*	
										7	2530	Col		
										8	2111	Col		
6	Rural	G/O	03.26.25	Urine	AMC	70234	167-A	O89:H21	120×	1	4,697,263	Chromosome		JBTFHI000000000
										2	138,815	IncFIB	*bla*_TEM-1B_, *tet(A)*, *mph(A)*, *aph(6)-Id*, *aph(3″)-Ib, dfrA14, sul2, gyrA* (S83L, D87N) and *parC* (S80I)	
										3	52,531	IncX1	*tet(A)*, *qnrS1*, *aph(3′)-Ia*, *dfrA14*	
										4	33,305	IncX4	*mcr-1.1*	
										5	5269	Col		
										6	4715	Col		

^a^: This patient carried two clonal isolates at 2.5-month interval; ^b^: Hospitalization ward: O, Outpatient settings; G/O, Gynecology/Obstetrics; G/E, Gastroenterology; P, Pediatrics; ^c^: m, month; d, Day; y, year; ^d^: Tx: treatment: Antibiotics received before the isolation of mcr-1-producing *E. coli* isolates; AMC, amoxicillin + clavulanic acid; CFX, cefixime; CTX, cefotaxime; DOX, doxycycline; GEN, gentamicin; MNZ, metronidazole; OFL, ofloxacin; ^e^: Achtman MLST scheme (https://enterobase.warwick.ac.uk/, accessed on 30 June 2026); ^f^: Depth, Average sequencing depth.

**Table 2:** Antimicrobial susceptibility patterns and minimum inhibitory concentrations (MICs) of *mcr-1*-producing *E. coli* strains using the broth microdilution method (Sensititre, ThermoFisher, LesUlis, France) and UMIC Cefiderocol^®^ kit (Biocentric, Bandol, France) for Cefiderocol.

Antibiotics		MIC of *E. coli* Isolates (mg/L)							Breakpoints (mg/L) ^a^
		70237	70240	70239	70242	70238	70233	70234	
B-lactams	Amoxicillin	**>32**	**>32**	**32**	**>32**	8	**>32**	**>32**	S ≤ 8, R > 8
	Amoxicillin + Clavulanic acid	**16**	**128**	**32**	**16**	8	**16**	**128**	S ≤ 8, R > 8
	Piperacillin	**>32**	**>32**	**32**	**>32**	≤4	**16**	**>32**	S ≤ 8, R > 8
	Piperacillin + Tazobactam	≤4	≤4	≤4	≤4	≤4	≤4	≤4	S ≤ 8, R > 8
	Ticarcillin	**>32**	**>32**	**>32**	**>32**	8	**>32**	**>32**	S ≤ 8, R > 8
	Temocillin	8	8	8	≤4	16	8	16	
	Cefoxitin	8	≤4	≤4	≤4	8	**32**	8	S ≤ 8, R > 8
	Ceftazidime	**16**	≤0.25	4	4	0.5	≤0.25	0.5	S ≤ 1, R > 4
	Ceftazidime + avibactam	≤0.25	≤0.25	≤0.25	≤0.25	≤0.25	≤0.25	≤0.25	S ≤ 8, R > 8
	Mecillinam	≤2	**>16**	**16**	4	**16**	4	**16**	S ≤ 8, R > 8
	Cefotaxime	**>8**	≤0.5	**>8**	**>8**	≤0.5	≤0.5	≤0.5	S ≤ 1, R > 2
	Ceftiofur	**>32**	≤1	**>32**	**>32**	≤1	≤1	≤1	S ≤ 2, I = 4, R ≥ 8
	Ceftobiprole	**>1**	≤0.06	**>1**	**>1**	≤0.06	0.12	0.12	S ≤ 0.25, R > 0.25
	Ceftaroline	**>2**	0.5	**>2**	**>2**	≤0.12	0.25	0.5	S ≤ 0.5, R > 0.5
	Cefalexin	**>128**	8	**>128**	**>128**	16	**32**	16	S ≤ 16, R > 16
	Aztreonam	**>16**	≤0.06	**8**	**8**	0.12	≤0.06	0.12	S ≤ 1, R > 4
	Aztreonam + avibactam	0.06	≤0.03	0.12	≤0.03	0.12	0.06	0.06	S ≤ 4, R > 4
	Cefepime	**>16**	≤0.5	**8**	4	≤0.5	≤0.5	≤0.5	S ≤ 1, R > 4
	Ceftolozane + tazobactam	**>8**	≤0.5	**>8**	**>8**	≤0.5	≤0.5	≤0.5	S ≤ 2, R > 2
	Cefiderocol (UMIC)	**16**	0.5	0.5	2	≤0.03	≤0.03	≤0.03	S ≤ 2, R > 2
	Ertapenem	≤0.25	≤0.25	≤0.25	≤0.25	≤0.25	≤0.25	≤0.25	S ≤ 0.5, R ≥ 0.5
	Imipenem	0.12	0.12	0.25	0.12	0.25	0.25	0.12	S ≤ 2, R ≥ 4
	Imipenem + relebactam	0.12	0.25	0.5	0.12	0.5	0.5	0.25	S ≤ 2, R > 2
	Meropenem	≤0.06	≤0.06	≤0.06	≤0.06	≤0.06	≤0.06	≤0.06	S ≤ 2, R > 8
	Meropenem + vaborbactam	≤0.03	≤0.03	≤0.03	≤0.03	≤0.03	≤0.03	≤0.03	S ≤ 8, R > 8
Fluoroquinolones	Norfloxacin	**>2**	**>2**	**2**	**>2**	0.12	**>2**	**>2**	S ≤ 0.5, R > 0.5
	Levofloxacin	**>4**	**1**	**4**	**4**	≤0.12	**>4**	**>4**	S ≤ 0.5, R ≥ 1
	Ciprofloxacin	**>2**	**1**	**2**	**>2**	≤0.06	**>2**	**>2**	S ≤ 0.125, R > 0.125
	Delafloxacin	**2**	**0.5**	**2**	**2**	≤0.03	**>2**	**>2**	S ≤ 0.125, R > 0.125
aminosides	Amikacin	≤4	≤4	≤4	≤4	≤4	≤4	≤4	S ≤ 8; R > 8
	Gentamicin	≤1	≤1	≤1	≤1	≤1	≤1	≤1	S ≤ 2, R > 2
	Tobramycin	≤1	2	≤1	≤1	≤1	≤1	≤1	S ≤ 2, R > 2
	Streptomycin ^b^	**32**	**16**	**32**	**>32**	≤4	**16**	**>32**	S ≤ 8; R > 16
	Apramycin ^b^	8	8	4	4	4	8	4	S ≤ 16; R > 16
	Neomycin ^b^	≤4	≤4	≤4	≤4	≤4	≤4	**>32**	S ≤ 8; R > 16
Cyclins	Tetracycline ^b^	**>16**	**>16**	**>16**	**>16**	**>16**	**>16**	**>16**	S ≤ 4, I = 8, R ≥ 16
	Tigecycline	≤0.25	≤0.25	≤0.25	≤0.25	≤0.25	0.5	≤0.25	S ≤ 0.5, R > 0.5
	Eravacycline	0.12	0.12	0.12	0.25	0.12	0.5	0.25	S ≤ 0.5, R > 0.5
Phenicol	Chloramphenicol ^b^	>16	8	8	>16	8	>16	16	no breakpoints
	Florfenicol ^b^	**>32**	4	8	**>32**	8	**>32**	8	S ≤ 8, R ≥ 32
various	Colistin (UMIC)	**8**	**4**	**4**	**4**	**4**	**4**	**4**	I ≤ 2; R > 2
	Fosfomycin + glucose-6-P	≤4	≤4	8	≤4	8	≤4	≤4	S ≤ 8, R > 8
	Nitrofurantoin	≤32	≤32	≤32	≤32	≤32	≤32	≤32	S ≤ 64, R > 64
	Trimethoprim	**>8**	**>8**	**>8**	**>8**	≤1	**>8**	**>8**	S ≤ 2; R > 2
	Sulfamethoxazole ^b^	**>512**	≤32	≤32	**>512**	≤32	**>512**	**>512**	S ≤ 64; R > 256
	Trimethoprim + sulfadiazine	**>8**	≤1	≤1	**>8**	≤1	**>8**	**>8**	S ≤ 0.5; R > 2

^a^: Broth microdilution breakpoints of the European Committee on Antimicrobial Susceptibility Testing (EUCAST: https://www.eucast.org) 2026 guidelines. Bold values are considered as resistant; ^b^: Broth microdilution breakpoints of the CA-SFM vétérinaire 2023 (https://www.sfm-microbiologie.org/2023/06/15/casfm-veterinaire-2023/; accessed on 30 June 2026).

## Data Availability

Whole-Genome Sequences are available at DDBJ/ENA/GenBank under the BioProject PRJNA1392031.

## Supplementary Materials

The following supporting information can be downloaded at https://www.mdpi.com/article/10.3390/antibiotics15080802/s1, Figure S1: Nucleotide sequence alignment of catecholate siderophore receptor *cirA* genes from *mcr-1* producing *Escherichia coli* isolates; Figure S2: Nucleotide sequence alignment of catecholate siderophore receptor *fiu* genes from *mcr-1* producing *Escherichia coli* isolates; Table S1: Clinical data of patients.
