# Supplementary material for: Reduced Susceptibility to Cefiderocol Among Clinical MCR-1-Producing Escherichia coli Isolates from Tunisia
Source: Antibiotics (Basel). 2026 Aug 18;15(8):802. doi: 10.3390/antibiotics15080802 (PMC13509618; doi:10.3390/antibiotics15080802)
Supplement: Supplementary file 1 [file antibiotics-15-00802-s001.zip › antibiotics-4442128-supplementary.pdf]

Supplemental Material

Article

Reduced Susceptibility to Cefiderocol Among Clinical MCR-1-Producing *Escherichia coli* Isolates from Tunisia

Nadia Jaidane [1,2], Thierry Naas [3,4,5,*], Souad Fayad [3], Pierre Châtre [6] Wejdene Mansour [1], Aymen Bouaziz [7,8], Pauline François [6], Laetitia Du Fraysseix [6], Bogdan I. Iorga [9], Nahed A. Al Laham [3,10], Lamia Tilouche [1], Farouk Barguellil [7,8] and Marisa Haenni [6]

Supplemental Figure S1 and S2

Figure S1: Nucleotide sequence alignment of catecholate siderophore receptor *cirA* genes from *mcr-1* producing *Escherichia coli* isolates from this study with that of the *Escherichia coli* ATCC 25922 strain (RefSeq; GenBank accession no. CP009072). Nucleotide changes are indicated by yellow highlighting. Dashes ('-') corresponds to deleted nucleotides. Underlined and bolded codons correspond to stop codons.

```
REFSEQ      ATGTTTAGGTTGAACCCTTTCGTACGGGTCGGGCTGTGTTTGTCCGCTATTTCTTGTGCA     60
70242       ATGTTTAGGTTGAACCCTTTCGTACGGGTCGGGCTGTGTTTGTCCGCTATTTCTTGTGCA     60
70239       ATGTTTAGGTTGAACCCTTTCGTACGGGTCGGGCTGTGTTTGTCCGCTATTTCTTGTGCA     60
70238       ATGTTTAGGTTGAACCCTTTCGTACGGGTCGGGCTGTGTTTGTCCGCTATTTCTTGTGCA     60
70234       ATGTTTAGGTTGAACCCTTTCGTACGGGTCGGGCTGTGTTTGTCCGCTATTTCTTGTGCA     60
70233       ATGTTTAGGTTGAACCCTTTCGTACGGGTCGGGCTGTGTTTGTCCGCTATTTCTTGTGCA     60
70237       ATGTTTAGGTTGAACCCTTTCGTACGGGTCGGGCTGTGTTTGTCCGCTATTTCTTGTGCA     60
70240       ATGTTTAGGTTGAACCCTTTCGTACGGGTCGGGCTGTGTTTGTCCGCTATTTCTTGTGCA     60
            ************************************************************

REFSEQ      TGGCCTGTGTTAGCGGTCGATGATGATGGCGAAACGATGGTTGTCACTGCATCTTCCGTT     120
70242       TGGCCTGTGTTAGCGGTCGATGATGATGGCGAAACGATGGTTGTCACTGCATCTTCCGTG     120
70239       TGGCCTGTGTTAGCGGTCGATGATGATGGCGAAACGATGGTTGTCACTGCATCTTCCGTG     120
70238       TGGCCTGTGTTAGCGGTCGATGATGATGGCGAAACGATGGTTGTCACTGCATCTTCCGTT     120
70234       TGGCCTGTGTTAGCGGTCGATGATGATGGCGAAACGATGGTTGTCACTGCATCTTCCGTG     120
70233       TGGCCTGTGTTAGCGGTCGATGACGATGGCGAAACGATGGTTGTCACTGCATCTTCCGTT     120
70237       TGGCCTGTGTTAGCGGTCGATGATGATGGCGAAACGATGGTTGTCACTGCATCTTCCGTT     120
70240       TGGCCTGTGTTAGCGGTCGATGATGATGGCGAAACGATGGTTGTCACTGCATCTTCCGTT     120
            ********************** ************************************ 

REFSEQ      GAACAAAACCTTAAAGATGCTCCCGCCAGTATCAGCGTCATTACCCAGGAAGACCTGCAG     180
70242       GAACAAAATCTTAAAGATGCACCTGCCAGTATCAGCGTCATTACCCAGGAAGACCTGCAG     180
70239       GAACAAAATCTTAAAGATGCACCTGCCAGTATCAGCGTCATTACCCAGGAAGACCTGCAG     180
70238       GAACAAAACCTTAAAGATGCACCTGCCAGTATCAGCGTCATTACCCAGGAAGACCTGCAG     180
70234       GAACAAAATCTTAAAGATGCACCTGCCAGTATCAGCGTCATTACCCAGGAAGACCTGCAG     180
70233       GAACAAAACCTTAAAGATGCACCCGCCAGTATCAGCGTCATTACCCAGGAAGACCTGCAG     180
70237       GAACAAAACCTTAAAGATGCACCCGCCAGTATCAGCGTCATTACCCAGGAAGACCTGCAG     180
70240       GAACAAAACCTTAAAGATGCACCCGCCAGTATCAGCGTCATTACCCAGGAAGACCTGCAG     180
            ******** *********** ** ************************************

REFSEQ      CGAAAACCGGTACAGAATCTGAAGGATGTCCTCAAAGAAGTGCCTGGCGTACAACTGACG     240
70242       CGAAAACCGGTACAGAATCTGAAGGATGTCCTCAAAGAAGTGCCTGGCGTACAACTGACG     240
70239       CGAAAACCGGTACAGAATCTGAAGGATGTCCTCAAAGAAGTGCCTGGCGTACAACTGACG     240
70238       CGAAAACCGGTACAGAATCTGAAGGATGTCCTCAAAGAAGTGCCTGGCGTACAACTGACG     240
70234       CGAAAACCGGTACAGAATCTGAAGGATGTCCTCAAAGAAGTGCCTGGCGTACAACTGACG     240
70233       CGAAAACCGGTACAGAATCTGAAGGATGTCCTCAAAGAAGTGCCTGGCGTACAACTGACG     240
70237       CGAAAACCGGTACAGAATCTGAAGGATGTCCTCAAAGAAGTGCCTGGCGTACAACTGACG     240
70240       CGAAAACCGGTACAGAATCTGAAGGATGTCCTCAAAGAAGTGCCTGGCGTACAACTGACG     240
            ************************************************************

REFSEQ      AACGAAGGGGATAACCGTAAGGGCGTAAGTATTCGTGGTCTGGACAGCAGCTACACCCTG     300
70242       AACGAAGGGGATAACCGTAAGGGCGTTAGTATTCGTGGTCTGGACAGCAGCTATACCCTG     300
70239       AACGAAGGGGATAACCGTAAGGGCGTTAGTATTCGTGGTCTGGACAGCAGCTATACCCTG     300
70238       AACGAAGGGGATAACCGTAAGGGCGTTAGTATTCGTGGTCTGGACAGCAGCTACACCCTG     300
70234       AACGAAGGGGATAACCGTAAGGGCGTTAGTATTCGTGGTCTGGACAGCAGCTATACCCTG     300
70233       AACGAAGGGGATAACCGTAAGGGCGTTAGTATTCGTGGTCTGGACAGCAGCTACACCCTG     300
70237       AACGAAGGGGATAACCGTAAGGGCGTTAGTATTCGTGGTCTGGACAGCAGCTACACCCTG     300
70240       AACGAAGGGGATAACCGTAAGGGCGTTAGTATTCGTGGTCTGGACAGCAGCTACACCCTG     300
            ************************** ************************** ******

REFSEQ      ATTCTTGTCGACGGTAAACGCGTTAACTCCCGCAATGCCGTCTTCCGCCACAATGATTTC     360
70242       ATTCTCGTCGACGGTAAACGCGTGAACTCCCGCAATGCCGTCTTCCGCCACAATGATTTC     360
70239       ATTCTCGTCGACGGTAAACGCGTGAACTCCCGCAATGCCGTCTTCCGCCACAATGATTTC     360
70238       ATTCTCGTCGACGGTAAACGCGTTAACTCCCGCAATGCCGTCTTCCGCCACAATGATTTC     360
70234       ATTCTCGTCGACGGTAAACGCGTGAACTCCCGCAATGCCGTCTTCCGCCACAATGATTTC     360
70233       ATTCTCGTCGACGGTAAACGCGTTAACTCCCGCAATGCCGTCTTCCGCCACAATGATTTC     360
```

```
70237       ATTCTTGTCGACGGTAAACGCGTTAACTCCCGCAATGCCGTCTTCCGCCACAATGATTTC      360
70240       ATTCTTGTCGACGGTAAACGCGTTAACTCCCGCAATGCCGTCTTCCGCCACAATGATTTC      360
            ***** ***************** ************************************

REFSEQ      GATCTGAACTGGATCCCGGTCGATTCCATCGAACGTATTGAAGTGGTTCGTGGCCCGATG      420
70242       GATCTGAACTGGATCCCGGTCGATTCCATCGAACGTATTGAAGTGGTCCGTGGCCCGATG      420
70239       GATCTGAACTGGATCCCGGTCGATTCCATCGAACGTATTGAAGTGGTCCGTGGCCCGATG      420
70238       GATCTGAACTGGATCCCGGTCGATTCCATCGAACGTATTGAAGTGGTCCGTGGCCCGATG      420
70234       GATCTGAACTGGATCCCGGTCGATTCCATCGAACGTATTGAAGTGGTCCGTGGCCCGATG      420
70233       GATCTGAACTGGATCCCGGTCGATTCCATCGAACGTATTGAAGTGGTTCGTGGCCCGATG      420
70237       GATCTGAACTGGATCCCGGTCGATTCCATCGAACGTATTGAAGTGGTCCGTGGCCCGATG      420
70240       GATCTGAACTGGATCCCGGTCGATTCCATCGAACGTATTGAAGTGGTCCGTGGCCCGATG      420
            *********************************************** ************

REFSEQ      TCGTCGCTGTACGGTTCCGATGCGCTCGGCGGTGTAGTGAATATCATCACCAAAAAAATC      480
70242       TCGTCGCTGTACGGTTCCGATGCGCTCGGCGGTGTAGTGAATATCATCACCAAAAAAATC      480
70239       TCGTCGCTGTACGGTTCCGATGCGCTCGGCGGTGTAGTGAATATCATCACCAAAAAAATC      480
70238       TCGTCGCTGTACGGTTCCGATGCGCTCGGCGGTGTAGTGAATATCATCACCAAAAAAATC      480
70234       TCGTCGCTGTACGGTTCCGATGCGCTCGGCGGTGTAGTGAATATCATCACCAAAAAAATC      480
70233       TCGTCGCTGTACGGCTCCGATGCGCTCGGCGGTGTAGTGAATATCATCACCAAAAAAATC      480
70237       TTGTCGCTGTACGGTTCCGATGCGCTCGGCGGTGTAGTGAATATCATCACCAAAAAAATC      480
70240       TCGTCGCTGTACGGTTCCGATGCGCTCGGCGGTGTAGTGAATATCATCACCAAAAAAATC      480
            * ************ *********************************************

REFSEQ      GGTCAGAAATGGTCGGGCACCGTTACCGTCGATACCACCGTTCAGGAACATCGCGATCGC      540
70242       GGTCAGAAATGGTCGGGTACCGTTACCGTCGATACCACCATTCAGGAACATCGCGATCGC      540
70239       GGTCAGAAATGGTCGGGTACCGTTACCGTCGATACCACCATTCAGGAACATCGCGATCGC      540
70238       GGTCAGAAATGGTCGGGTACCGTTACCGTCGATACCACCATTCAGGAACATCGCGATCGC      540
70234       GGTCAGAAATGGTCGGGTACCGTTACCGTCGATACCACCATTCAGGAACATCGCGATCGC      540
70233       GGTCAGAAATGGTCGGGTACCGTTACCGTCGATACCACCATTCAGGAACATCGCGATCGC      540
70237       GGTCAGAAATGGTCGGGTACCGTTACCGTCGATACCACCATTCAGGAACATCGCGATCGC      540
70240       GGTCAGAAATAGTCGGGTACCGTTACCGTCGATACCACCATTCAGGAACATCGCGATCGC      540
            ********** ****** ********************* ********************

REFSEQ      GGTGATACCTATAACGGTCAATTCTTTACCAGCGGACCATTAATTGACGGCGTGCTGGGA      600
70242       GGTGACACCTATAACGGTCAGTTCTTTACCAGTGGACCATTAATTGATGGTGTGCTGGGA      600
70239       GGTGACACCTATAACGGTCAGTTCTTTACCAGTGGACCATTAATTGATGGTGTGCTGGGA      600
70238       GGTGATACCTATAACGGTCAGTTCTTTACCAGCGGACCATTAATTGACGGTGTGCTGGGA      600
70234       GGTGACACCTATAACGGTCAGTTCTTTACCAGTGGACCATTAATTGATGGTGTGCTGGGA      600
70233       GGTGATACCTATAACGGTCAGTTCTTTACCAGTGGACCATTAATTGATGGTGTGCTGGGA      600
70237       -GTGATACCTATAACGGTCAGTTCTTTACCAGTGGACCATTAATTGATGGTGTGCTGGGA      599
70240       GGTGATACCTATAACGGTCAGTTCTTTACCAGTGGACCATTAATTGATGGTGTGCTGGGA      600
             **** *************** *********** ************** ** *********

REFSEQ      ATGAAAGCTTACGGCAGCCTGGCAAAACGTGAAAAGGATGACCCGCAAAACTCAACGACC      660
70242       ATGAAAGCTTACGGCAGCCTGGCAAAACGTGAAAAGGATGACCCGCAAAACTCAACGACC      660
70239       ATGAAAGCTTACGGCAGCCTGGCAAAACGTGAAAAGGATGACCCGCAAAACTCAACGACC      660
70238       ATGAAAGCTTACGGCAGCCTGGCAAAACGTGAAAAGGATGACCCGCAAAACTCAACGACC      660
70234       ATGAAAGCTTACGGCAGCCTGGCAAAACGTGAAAAGGATGACCCGCAAAACTCAACGACC      660
70233       ATGAAAGCTTACGGCAGCCTGGCAAAACGTGAAAAGGATGACCCGCAAAACTCAACGACC      660
70237       ATGAAAGCTTACGGCAGCCGGGCAAAACGTGAAAAGGATGACCCGCAAAACTCAACGACC      659
70240       ATGAAAGCTTACGGCAGCCTGGCAAAACGTGAAAAGGATGACCCGCAAAACTCAACGACC      660
            ******************* ****************************************

REFSEQ      ACCGATACCGGAGAAACGCCGCGTATTGAAGGATTCTCCAGCCGCGACGGCAATGTCGAA      720
70242       ACCGATACCGGAGAAACGCCGCGTATTGAAGGATTCTCCAGCCGCGACGGCAATGTCGAA      720
70239       ACCGATACCGGAGAAACGCCGCGTATTGAAGGATTCTCCAGCCGCGACGGCAATGTCGAA      720
70238       ACCGATACCGGAGAAACGCCGCGTATTGAAGGATTCTCCAGCCGCGACGGCAATGTCGAA      720
70234       ACCGATACCGGAGAAACGCCGCGTATTGAAGGATTCTCCAGCCGCGACGGCAATGTCGAA      720
70233       ACCGATACCGGAGATACGCCGCGTATTGAAGGATTCTCCAGCCGCGACGGCAATGTCGAA      720
70237       ACCGATACCGGAGAAACGCCGCGTATTGAAGGATTCTCCAGCCGCGACGGCAATGTCGAA      719
70240       ACCGATACCGGAGATACGCCGCGTATTGAAGGATTCTCCAGCCGCGACGGCAATGTCGAA      720
```

```
             ************** *********************************************

REFSEQ       TTTGCCTGGACACCGAATCAAAATCACGATTTTACTGCCGGATACGGTTTCGACCGTCAG     780
70242        TTTGCCTGGACACCGAATCAAAATCACGATTTTACTGCCGGATACGGTTTCGACCGTCAG     780
70239        TTTGCCTGGACACCGAATCAAAATCACGATTTTACTGCCGGATACGGTTTCGACCGTCAG     780
70238        TTTGCCTGGACACCGAATCAAAATCACGATTTTACTGCCGGATACGGTTTCGACCGTCAG     780
70234        TTTGCCTGGACACCGAATCAAAATCACGATTTTACTGCCGGATACGGTTTCGACCGTCAG     780
70233        TTTGCCTGGACACCGAATCAAAATCACGATTTTACTGCCGGATACGGTTTCGACCGTCAG     780
70237        TTTGCCTGGACACCGAATCAAAATCACGATTTTACTGCCGGATACGGTTTCGACCGTCAG     779
70240        TTTGCCTGGACACCGAATCAAAATCACGATTTTACTGCCGGATACGGTTTCGACCGTCAG     780
             ************************************************************

REFSEQ       GATCGTGATTCCGACTCGCTGGACAAAAACCGCCTGGAACGCCAGAACTACTCCGTCAGC     840
70242        GATCGTGATTCCGACTCGCTGGACAAAAACCGCCTGGAACGCCAGAACTACTCCGTCAGC     840
70239        GATCGTGATTCCGACTCGCTGGACAAAAACCGCCTGGAACGCCAGAACTACTCCGTCAGC     840
70238        GATCGTGATTCCGACTCGCTGGACAAAAACCGCCTGGAACGCCAGAACTACTCCGTCAGC     840
70234        GATCGTGATTCCGACTCGCTGGACAAAAACCGCCTGGAACGCCAGAACTACTCCGTCAGC     840
70233        GATCGTGATTCCGACTCGCTGGACAAAAACCGCCTGGAACGCCAGAACTACTCCGTCAGC     840
70237        GATCGTGATTCCGACTCGCTGGACAAAAACCGCCTGGAACGCCAGAACTACTCCGTCAGC     839
70240        GATCGTGATTCCGACTCGCTGGACAAAAACCGCCTGGAACGCCAGAACTACTCCGTCAGC     840
             ************************************************************

REFSEQ       CATAATGGGCGTTGGGATTACGGCACCAGCGAACTGAAATACTACGGTGAGAAAGTCGAG     900
70242        CATAATGGGCGTTGGGATTACGGCACCAGCGAACTGAAATACTACGGTGAGAAAGTCGAG     900
70239        CATAATGGGCGTTGGGATTACGGCACCAGCGAACTGAAATACTACGGTGAGAAAGTCGAG     900
70238        CATAATGGGCGTTGGGATTACGGCACCAGCGAACTGAAATACTACGGTGAGAAAGTCGAG     900
70234        CATAATGGGCGTTGGGATTACGGCACCAGCGAACTGAAATACTACGGTGAGAAAGTCGAG     900
70233        CATAATGGGCGTTGGGATTACGGCACCAGCGAACTGAAATACTACGGTGAGAAAGTCGAG     900
70237        CATAATGGGCGTTGGGATTACGGCACCAGCGAACTGAAATACTACGGTGAGAAAGTCGAG     899
70240        CATAATGGGCGTTGGGATTACGGCACCAGCGAACTGAAATACTACGGTGAGAAAGTCGAG     900
             ************************************************************

REFSEQ       AACAAAAACCCTGGCAACAGCAGCCCGATAACTTCCGAAAGCAATACGGTCGACGGCAAA     960
70242        AACAAAAACCCTGGCAACAGCAGCCCGATAACTTCCGAAAGCAATACGGTCGACGGCAAA     960
70239        AACAAAAACCCTGGCAACAGCAGCCCGATAACTTCCGAAAGCAATACGGTCGACGGCAAA     960
70238        AACAAAAACCCTGGCAACAGCAGCCCGATAACTTCCGAAAGCAATACGGTCGACGGCAAA     960
70234        AACAAAAACCCTGGCAACAGCAGCCCGATAACTTCCGAAAGCAATACGGTCGACGGCAAA     960
70233        AACAAAAACCCTGGCAACAGCAGCCCGATAACTTCCGAAAGCAATACGGTCGACGGCAAA     960
70237        AACAAAAACCCTGGCAACAGCAGCCCGATAACTTCCGAAAGCAATACGGTCGACGGCAAA     959
70240        AACAAAAACCCTGGCAACAGCAGCCCGATAACTTCCGAAAGCAATACGGTCGACGGCAAA     960
             ************************************************************

REFSEQ       TACACGTTGCCGCTGACGGCGATTAATCAGTTTCTCACGGTTGGCGGTGAATGGCGTCAC     1020
70242        TACACGTTGCCGCTGACGGCGATTAATCAGTTTCTCACGGTTGGCGGTGAATGGCGTCAC     1020
70239        TACACGTTGCCGCTGACGGCGATTAATCAGTTTCTCACGGTTGGCGGTGAATGGCGTCAC     1020
70238        TACACGTTGCCGCTGACGGCGATTAATCAGTTTCTCACGGTTGGCGGTGAATGGCGTCAC     1020
70234        TACACGTTGCCGCTGACGGCGATTAATCAGTTTCTCACGGTTGGCGGTGAATGGCGTCAC     1020
70233        TACACCTTGCCGCTGACGGCGATTAATCAGTTTCTCACGGTTGGCGGTGAATGGCGTCAC     1020
70237        TACACGTTGCCGCTGACGGCGATTAATCAGTTTCTCACGGTTGGCGGTGAATGGCGTCAC     1019
70240        TACACGTTGCCGCTGACGGCGATTAATCAGTTTCTCACGGTTGGCGGTGAATGGCGTCAC     1020
             ***** ******************************************************

REFSEQ       GACAAACTTAGCGATGCGGTGAACCTGACCGGGGGAACCAGCTCCAAAACGTCTGCCAGC     1080
70242        GACAAACTTAGCGATGCGGTGAACCTGACCGGGGGAACCAGCTCCAAAACGTCTGCCAGC     1080
70239        GACAAACTTAGCGATGCGGTGAACCTGACCGGGGGAACCAGCTCCAAAACGTCTGCCAGC     1080
70238        GACAAACTTAGCGATGCGGTGAACCTGACTGGGGGAACCAGCTCCAAAACGTCTGCCAGC     1080
70234        GACAAACTTAGCGATGCGGTGAACCTGACCGGGGGAACCAGCTCCAAAACGTCTGCCAGC     1080
70233        GACAAACTTAGTGATGCGGTGAACCTGACCGGGGGAACCAGCTCCAAAACGTCTGCCAGC     1080
70237        GACAAACTTAGCGATGCGGTGAACCTGACCGGGGGAACCAGCTCCAAAACGTCTGCCAGC     1079
70240        GACAAACTTAGCGATGCGGTGAACCTGACCGGGGGAACCAGCTCCAAAACGTCTGCCAGC     1080
             *********** ***************** ******************************
```

```
REFSEQ      CAGTACGCGCTGTTTGTGGAAGATGAATGGCGGATCTTCGAGCCGCTGGCGCTGACGACC    1140
70242       CAGTACGCGCTGTTTGTGGAAGATGAATGGCGGATCTTCGAGCCGCTGGCGCTGACGACC    1140
70239       CAGTACGCGCTGTTTGTGGAAGATGAATGGCGGATCTTCGAGCCGCTGGCGCTGACGACC    1140
70238       CAGTACGCGCTGTTTGTGGAAGATGAATGGCGGATCTTCGAGCCGCTGGCGCTGACGACC    1140
70234       CAGTACGCGCTGTTTGTGGAAGATGAATGGCGGATCTTCGAGCCGCTGGCGCTGACGACC    1140
70233       CAGTACGCGCTGTTTGTGGAAGATGAATGGCGGATCTTCGAGCCGCTGGCGCTGACGACC    1140
70237       CAGTACGCGCTGTTTGTGGAAGATGAATGGCGAATCTTCGAGCCGCTGGCGCTGACGACC    1139
70240       CAGTACGCGCTGTTTGTGGAAGATGAATGGCGGATCTTCGAGCCGCTGGCGCTGACGACC    1140
            ******************************** ***************************

REFSEQ      GGCGTGCGTATGGACGATCACGAAACCTACGGTGAACACTGGAGTCCGCGTGCCTACCTG    1200
70242       GGCGTGCGTATGGACGATCACGAAACCTACGGTGAACACTGGAGTCCGCGTGCCTACCTG    1200
70239       GGCGTGCGTATGGACGATCACGAAACCTACGGTGAACACTGGAGTCCGCGTGCCTACCTG    1200
70238       GGCGTACGTATGGACGATCATGAAACCTACGGTGAACACTGGAGTCCGCGTGCCTACCTG    1200
70234       GGCGTGCGTATGGACGATCACGAAACCTACGGTGAACACTGGAGTCCGCGTGCCTACCTG    1200
70233       GGCGTACGTATGGACGATCATGAAACCTACGGTGAACACTGGAGTCCGCGTGCCTACCTG    1200
70237       GGCGTACGTATGGACGATCATGAAACCTACGGTGAACACTGGAGTCCGCGTGCCTACCTG    1199
70240       GGCGTGCGTATGGACGATCACGAAACCTACGGTGAACACTGGAGTCCGCGTGCCTACCTG    1200
            ***** ************** ***************************************

REFSEQ      GTTTATAACGCCACCGACACCGTAACGGTGAAAGGGGGCTGGGCGACGGCATTTAAAGCC    1260
70242       GTTTATAACGCCACCGACACCGTAACGGTGAAAGGGGGCTGGGCGACGGCATTTAAAGCA    1260
70239       GTTTATAACGCCACCGACACCGTAACGGTGAAAGGGGGCTGGGCGACGGCATTTAAAGCA    1260
70238       GTTTATAACGCCACCGACACCGTAACGGTAAAAGGGGGCTGGGCGACGGCATTTAAAGCA    1260
70234       GTTTATAACGCCACCGACACCGTAACGGTGAAAGGGGGCTGGGCGACGGCATTTAAAGCA    1260
70233       GTTTATAACGCCACCGACACCGTAACGGTGAAAGGGGGCTGGGCGACGGCATTTAAAGCA    1260
70237       GTTTATAACGCCACCGACACCGTAACGGTGAAAGGGGGCTGGGCGACGGCATTTAAAGCG    1259
70240       GTTTATAACGCCACCGACACCGTAACGGTGAAAGGGGGCTGGGCGACGGCATTTAAAGCA    1260
            ***************************** ***************************** 

REFSEQ      CCTTCTCTGTTGCAACTTAGCCCTGACTGGACGAGCAATTCCTGCCGTGGCGCATGTAAG    1320
70242       CCTTCTCTGTTGCAACTTAGCCCTGACTGGACGAGCAATTCCTGCCGTGGCGCATGTAAG    1320
70239       CCTTCTCTGTTGCAACTTAGCCCTGACTGGACGAGCAATTCCTGCCGTGGCGCATGTAAG    1320
70238       CCTTCTCTGTTGCAACTTAGCCCTGACTGGACGAGCAATTCTTGCCGTGGCGCATGTAAG    1320
70234       CCTTCTCTGTTGCAACTTAGCCCTGACTGGACGAGCAATTCCTGCCGTGGCGCATGTAAG    1320
70233       CCTTCTCTGTTGCAACTTAGCCCTGACTGGACGAGCAATTCCTGCCGTGGCGCATGTAAG    1320
70237       CCTTCTCTGTTGCAACTTAGCCCTGACTGGACGAGCAATTCCTGCTGTGGCGCATGTAAG    1319
70240       CCTTCTCTGTTGCAACTTAGCCCTGACTGGACGAGCAATTCCTGCCGTGGCGCATGTAAG    1320
            ***************************************** *** **************

REFSEQ      ATTGTGGGTAGCCCGGATCTGAAACCAGAAACCAGCGAAAGTTGGGAGCTGGGGCTTTAC    1380
70242       ATTGTGGGTAGCCCGGATCTGAAACCAGAAACCAGCGAAAGTTGGGAGCTGGGGCTTTAC    1380
70239       ATTGTGGGTAGCCCGGATCTGAAACCAGAAACCAGCGAAAGTTGGGAGCTGGGGCTTTAC    1380
70238       ATTGTGGGTAGCCCGGATCTGAAACCAGAAACGAGCGAAAGTTGGGAGCTGGGGCTTTAC    1380
70234       ATTGTGGGTAGCCCGGATCTGAAACCAGAAACCAGCGAAAGTTGGGAGCTGGGGCTTTAC    1380
70233       ATTGTGGGTAGCCCGGATCTGAAACCAGAAACCAGCGAAAGTTGGGAGCTGGGGCTTTAC    1380
70237       ATTGTGGGTAGCCCGGATCTGAAACCAGAAACCAGCGAAAGTTGGGAGCTGGGGCTTTAC    1379
70240       ATTGTGGGTAGCCCGGATCTGAAACCAGAAACCAGCGAAAGTTGGGAGCTGGGGCTTTAC    1380
            ******************************** ***************************

REFSEQ      TACATGGGTGAAGAAGGCTGGCTGGAAGGGGTTGAATCCAGCGTTACCGTTTTCCGTAAC    1440
70242       TACATGGGTGAAGAAGGCTGGCTGGAAGGGGTTGAATCCAGCGTTACCGTTTTCCGTAAC    1440
70239       TACATGGGTGAAGAAGGCTGGCTGGAAGGGGTTGAATCCAGCGTTACCGTTTTCCGTAAC    1440
70238       TACATGGGTGAAGAAGGCTGGCTGGAAGGGGTTGAATCCAGCGTTACCGTTTTCCGTAAC    1440
70234       TACATGGGTGAAGAAGGCTGGCTGGAAGGGGTTGAATCCAGCGTTACCGTTTTCCGTAAC    1440
70233       TACATGGGCGAAGAAGGCTGGCTGGAAGGGGTTGAATCCAGCGTTACCGTTTTCCGTAAC    1440
70237       TACATGGGTGAAGAAGGCTGGCTGGAAGGGGTTGAATCCAGCGTTACCGTTTTCCGTAAC    1439
70240       TACATGGGTGAAGAAGGCTGGCTGGAAGGGGTTGAATCCAGCGTTACCGTTTTCCGTAAC    1440
            ******** ***************************************************

REFSEQ      GATGTGAAAGATCGTATCAGCATTAGCCGTACGTCTGACGTCAATGCTGCACCGGGCTAC    1500
70242       GATGTGAAAGATCGTATCAGCATCAGCCGTACGTCTGACGTCAACGCTGCACCGGGCTAC    1500
```

```
70239      GATGTGAAAGATCGTATCAGCATCAGCCGTACGTCTGACGTCAACGCTGCACCGGGCTAC      1500
70238      GATGTGAAAGATCGTATCAGCATCAGCCGTACGTCTGACGTCAATGCTGCACCAGGCTAC      1500
70234      GATGTGAAAGATCGTATCAGCATCAGCCGTACGTCTGACGTCAACGCTGCACCGGGCTAC      1500
70233      GATGTGAAAGATCGTATCAGCATCAGCCGTACGTCTGACGTCAATGCTGCACCAGGCTAC      1500
70237      GATGTGAAAGATCGTATCAGCATCAGCCGTACGTCTGACGTCAATGCTGCACCGGGCTAC      1499
70240      GATGTGAAAGATCGTATCAGCATCAGCCGTACGTCTGACGTCAATGCTGCACCGGGCTAC      1500
           *********************** ******************** ******** ******

REFSEQ     CAAAACTTTGTCGGTTTTGAGACGGGCGCTAACGGACGGCGCATACCGGTATTTAGCTAC      1560
70242      CAAAACTTTGTTGGTTTTGAGACGGGCGCTAACGGACGGCGCATACCGGTATTTAGCTAC      1560
70239      CAAAACTTTGTTGGTTTTGAGACGGGCGCTAACGGACGGCGCATACCGGTATTTAGCTAC      1560
70238      CAAAACTTTGTTGGTTTTACAGCGAA------------CGGTGTGCCGGTATTTAGCTAC      1548
70234      CAAAACTTTGTTGGTTTTGAGACGGGCGCTAACGGACGGCGCATACCGGTATTTAGCTAC      1560
70233      CAAAACTTTGTCGGTTTTGAGACGGGCGCTAACGGACGGCGCATACCGGTATTTAGCTAC      1560
70237      CAAAACTTTGTTGGTTTTACAGCGAA------------CGGTGTGCCGGTATTTAGCTAC      1547
70240      CAAAACTTTGTTGGTTTTACAGCGAA------------CGGTGTGCCGGTATTTAGCTAC      1548
           *********** ******    **                *  * ***************

REFSEQ     TACAACGTTAACAAAGCTCGTATTCAGGGCGTGGAAACCGAACTGAAAATTCCGTTCAAC      1620
70242      TACAACGTTAACAAAGCTCGTATTCAGGGCGTGGAAACCGAACTGAAAATTCCGTTCAAC      1620
70239      TACAACGTTAACAAAGCTCGTATTCAGGGCGTGGAAACCGAACTGAAAATTCCGTTCAAC      1620
70238      TACAACGTTAACAAAGCTCGTATTCAGGGCGTGGAAACCGAACTGAAAATTCCGTTCAAC      1608
70234      TACAACGTTAACAAAGCTCGTATTCAGGGCGTGGAAACCGAACTGAAAATTCCGTTCAAC      1620
70233      TACAACGTTAACAAAGCTCGTATTCAGGGCGTGGAAACCGAACTGAAAATTCCGTTCAAC      1620
70237      TACAACGTTAACAAAGCACGTATTCAGGGCGTGGAAACCGAACTGAAAATTCCGTTCAAC      1607
70240      TACAACGTTAACAAAGCTCGTATTCAGGGCGTGGAAACCGAACTGAAAATTCCGTTCAAC      1608
           ***************** ******************************************

REFSEQ     GATGAATGGAAACTGTCGATCAACTACACCTACAACGATGGTCGTGATGTCAGCAACGGC      1680
70242      GATGAATGGAAACTGTCGATCAACTACACCTACAACGATGGTCGTGATGTCAGCAACGGC      1680
70239      GATGAATGGAAACTGTCGATCAACTACACCTACAACGATGGTCGTGATGTCAGCAACGGC      1680
70238      GATGAATGGAAACTGTCACTCAACTACACCTACAACGATGGTCGTGATGTCAGCAACGGC      1668
70234      GATGAATGGAAACTGTCGATCAACTACACCTACAACGATGGTCGTGATGTCAGCAACGGC      1680
70233      GATGAATGGAAACTGTCGATCAACTACACCTACAACGATGGTCGTGATGTCAGCAACGGC      1680
70237      GATGAATGGAAACTGTCACTCAACTACACCTACAACGATGGTCGTGATGTCAGCAACGGC      1667
70240      GATGAATGGAAACTGTCACTCAACTACACCTACAACGATGGTCGTGATGTCAGCAACGGC      1668
           *****************  *****************************************

REFSEQ     GAAAACAAACCGCTATCCGATCTGCCGTTCCATACTGCTAACGGTACGCTGGACTGGAAA      1740
70242      GAAAACAAACCGCTATCCGATCTGCCGTTCCATACTGCTAACGGTACGCTGGACTGGAAA      1740
70239      GAAAACAAACCGCTATCCGATCTGCCGTTCCATACTGCTAACGGTACGCTGGACTGGAAA      1740
70238      GAAAATAAACCTTTATCCGATCTGCCGTTCCATACCGCTAACGGTACGCTGGACTGGAAA      1728
70234      GAAAACAAACCGCTATCCGATCTGCCGTTCCATACTGCTAACGGTACGCTGGACTGGAAA      1740
70233      GAAAACAAACCGCTATCCGATCTGCCGTTCCATACTGCTAACGGTACGCTGGACTGGAAA      1740
70237      GAAAACAAACCGCTATCCGATCTGCCGTTCCATACCGCTAACGGTACGCTGGACTGGAAA      1727
70240      GAAAACAAACCGCTATCCGATCTGCCGTTCCATACCGCTAACGGTACGCTGGACTGGAAA      1728
           ***** *****  ********************** ************************

REFSEQ     CCGCTGGCGCTGGAAGACTGGTCATTCTATGTTTCTGGTCACTATACCGGGCAGAAACGC      1800
70242      CCGCTGGCGCTGGAAGACTGGTCATTCTATGTTTCTGGGCACTATACCGGGCAGAAACGC      1800
70239      CCGCTGGCGCTGGAAGACTGGTCATTCTATGTTTCTGGGCACTATACCGGGCAGAAACGC      1800
70238      CCGCTGGCGCTGGAAGACTGGTCATTCTATGTTTCTGGTCACTATACCGGGCAGAAACGC      1788
70234      CCGCTGGCGCTGGAAGACTGGTCATTCTATGTTTCTGGGCACTATACCGGGCAGAAACGC      1800
70233      CCGCTGGCGCTGGAAGACTGGTCATTCTATGTTTCTGGTCACTATACCGGGCAGAAACGC      1800
70237      CCGCTGGCACTGGAAGACTGGTCATTCTATGTTTCTGGTCACTATACCGGGCAGAAACGA      1787
70240      CCGCTGGCGCTGGAAGACTGGTCATTCTATGTTTCTGGTCACTATACCGGGCAGAAACGA      1788
           ******** ***************************** ********************

REFSEQ     GCCGACAGCGCGACGGCTAAAACACCGGGCGGTTATACCATCTGGAATACCGGCGCGGCC      1860
70242      GCCGACAGCGCGACGGCTAAAACACCGGGCGGTTATACCATCTGGAATACCGGCGCGGCC      1860
70239      GCCGACAGCGCGACGGCTAAAACACCGGGCGGTTATACCATCTGGAATACCGGCGCGGCC      1860
70238      GCCGACAGCGCGACGGCTAAAACACCGGGCGGTTATACCATCTGGAATACCGGTGCGGCC      1848
```

```
70234         GCCGACAGCGCGACGGCTAAAACACCGGGCGGTTATACCATCTGGAATACCGGCGCGGCC        1860
70233         GCCGACAGCGCGACGGCTAAAACACCGGGCGGTTATACCATCTGGAATACCGGCGCGGCC        1860
70237         GCCGACAGCGCGACGGCTAAAACACCGGGCGGTTATACCATCTGGAATACCGGCGCGGCC        1847
70240         GCCGACAGCGCGACGGCTAAAACACCTAGCGGTTATACCATCTGGAATACCGGCGCGGCC        1848
              **************************  ************************* ******

REFSEQ        TGGCAGGTGACTAAAGACGTCAAACTGCGCGCAGGCGTGCTGAACCTTGGCGACAAGGAT        1920
70242         TGGCAGGTGACTAAAGACGTCAAACTGCGCGCAGGCGTGCTGAACCTTGGCGACAAGGAT        1920
70239         TGGCAGGTGACTAAAGACGTCAAACTGCGCGCAGGCGTGCTGAACCTTGGCGACAAGGAT        1920
70238         TGGCAGGTGACTAAAGACGTCAAACTGCGCGCAGGCGTGCTGAACCTTGGCGACAAGGAT        1908
70234         TGGCAGGTGACTAAAGACGTCAAACTGCGCGCAGGCGTGCTGAACCTTGGCGACAAGGAT        1920
70233         TGGCAGGTGACTAAAGACGTCAAACTGCGCGCAGGCGTGCTGAACCTTGGCGACAAGGAT        1920
70237         TGGCAGGTGACTAAAGACGTCAAACTGCGCGCAGGCGTGCTGAACCTTGGCGACAAGGAT        1907
70240         TGGCAGGTGACTAAAGACGTCAAACTGCGCGCAGGCGTGCTGAACCTTGGCGACAAGGAT        1908
              ************************************************************

REFSEQ        CTCAGTCGTGACGACTACAGCTATAACGAAGACGGACGTCGTTACTTTATGGCAGTGGAT        1980
70242         CTCAGTCGTGACGACTACAGCTATAACGAAGACGGACGTCGTTACTTTATGGCAGTGGAT        1980
70239         CTCAGTCGTGACGACTACAGCTATAACGAAGACGGACGTCGTTACTTTATGGCAGTGGAT        1980
70238         CTCAGTCGTGACGACTACAGCTATAACGAAGACGGACGTCGTTACTTTATGGCAGTGGAT        1968
70234         CTCAGTCGTGACGACTACAGCTATAACGAAGACGGACGTCGTTACTTTATGGCAGTGGAT        1980
70233         CTCAGTCGTGACGACTACAGCTATAACGAAGACGGACGTCGTTACTTTATGGCAGTGGAT        1980
70237         CTCAGTCGTGACGACTACAGCTATAACGAAGACGGACGTCGTTACTTTATGGCAGTGGAT        1967
70240         CTCAGTCGTGACGACTACAGCTATAACGAAGACGGACGTCGTTACTTTATGGCAGTGGAT        1968
              ************************************************************

REFSEQ        TATCGCTTCTGA        1992
70242         TATCGCTTCTGA        1992
70239         TATCGCTTCTGA        1992
70238         TATCGCTTCTGA        1980
70234         TATCGCTTCTGA        1992
70233         TATCGCTTCTGA        1992
70237         TATCGCTTCTGA        1979
70240         TATCGCTTCTGA        1980
              ************
```

Figure S2: Nucleotide sequence alignment of catecholate siderophore receptor *fiuA* genes from *mcr-1* producing *Escherichia coli* isolates from this study with that of the *Escherichia coli* ATCC 25922 strain (RefSeq; GenBank accession no. CP009072). Nucleotide changes are indicated by yellow highlighting. Dashes ('-') corresponds to deleted nucleotides. Underlined and bolded codons correspond to stop codons.

```
Fiu_refseq      ATGGAAAACAATCGCAATTTCCCTGCCAGACAATTTCATTCGCTCACGTTCTTTGCCGGT   60
70233           ATGGAAAACAATCGCAATTTCCCTGCCAGACAATTTCATTCGCTCACGTTCTTTGCCGGT   60
70238           ATGGAAAACAATCGCAATTTCCCTGCCAGACAATTTCATTCGCTCACGTTCTTTGCCGGT   60
70234           ATGGAAAACAATCGCAATTTCCCTGCCAGACAATTTCATTCGCTCACGTTCTTTGCCGGT   60
70239           ATGGAAAACAATCGCAATTTCCCTGCCAGACAATTTCATTCGCTCACGTTCTTTGCCGGT   60
70242           ATGGAAAACAATCGCAATTTCCCTGCCAGACAATTTCATTCGCTCACGTTCTTTGCCGGT   60
70237           ATGGAAAACAATCGCAATTTCCCTGCCAGACAATTTCATTCGCTCACGTTCTTTGCCGGT   60
70240           ATGGAAAACAATCGCAATTTCCCTGCCAGACAATTTCATTCGCTCACGTTCTTTGCCGGT   60
                ************************************************************

Fiu_refseq      CTTTGTATTGGTATTACGCCTGTGGCACAGGCACTCGCTGCCGAAGGGCAAGCTAACGCG   120
70233           CTTTGTATTGGCATCACGCCTGTGGCTCAGGCACTCGCCGCCGAAGGGCAAACTAACGCG   120
70238           CTTTGTATTGGCATCACGCCTGTGGCTCAGGCTCTCGCCGCCGAAGGGCAAACTAACGCG   120
70234           CTTTGTATTGGCATCACGCCTGTGGCTCAGGCACTCGCCGCCGAAGGGCAAACTAACGCG   120
70239           CTTTGTATTGGCATCACGCCTGTGGCTCAGGCACTCGCCGCCGAAGGGCAAACTAACGCG   120
70242           CTTTGTATTGGCATCACGCCTGTGGCTCAGGCACTCGCCGCCGAAGGGCAAACTAACGCG   120
70237           CTTTGTATTGGTATTACGCCTGTGGCGCAGGCACTCGCTGCCGAAGGGCAAGCTAACGCG   120
70240           CTTTGTATTGGCATCACGCCTGTGGCTCAGGCACTCGCCGCCGAAGGGCAAGCAAACGCG   120
                *********** ** *********** ***** ***** ************ * ******

Fiu_refseq      GATGACACGCTGGTTGTCGAAGCATCAACGCCTTCGCTTTATGCGCCACAACAATCTGCC   180
70240           GATGACACGCTGGTTGTCGAAGCATCAACGCCTTCGCTTTATGCGCCACAACAATCTGCC   180
70237           GATGACACGCTGGTTGTCGAAGCATCAACGCCTTCGCTTTATGCGCCACAACAATCTGCC   180
70233           GATGACACGCTGGTTGTCGAAGCATCAACGCCTTCGCTTTATGCGCCACAACAATCTGCA   180
70238           GATGACACGCTGGTTGTCGAAGCATCAACGCCTTCGCTTTATGCGCCACAACAATCTGCC   180
70234           GATGACACGCTGGTTGTCGAAGCATCAACGCCTTCGCTTTATGCGCCACAACAATCTGCC   180
70239           GATGACACGCTGGTTGTCGAAGCATCAACGCCTTCGCTTTATGCGCCACAACAATCTGCC   180
70242           GATGACACGCTGGTTGTCGAAGCATCAACGCCTTCGCTTTATGCGCCACAACAATCTGCC   180
                *********************************************************** 

Fiu_refseq      GATCCGAAATTCTCGCGTCCGGTAGCGGATACTACCCGCACGATGACGGTGATTTCTGAA   240
70233           GATCCGAAATTCTCGCGTCCGGTAGCGGATACTACCCGCACGATGACGGTGATTTCTGAA   240
70238           GATCCGAAATTCTCGCGTCCGGTAGCGGATACTACCCGCACGATGACGGTGATTTCTGAA   240
70234           GATCCGAAATTCTCGCGTCCGGTAGCGGATACTACCCGCACGATGACGGTAATTTCTGAA   240
70239           GATCCGAAATTCTCGCGTCCGGTAGCGGATACTACCCGCACGATGACGGTGATTTCTGAA   240
70242           GATCCGAAATTCTCGCGTCCGGTAGCGGATACTACCCGCACGATGACGGTGATTTCTGAA   240
70237           GATCCGAAATTCTCGCGTCCGGTAGCGGATACTACCCGCACGATGACGGTGATTTCTGAA   240
70240           GATCCGAAATTCTCGCGTCCGGTAGCGGATACTACCCGCACGATGACGGTGATTTCTGAA   240
                ************************************************** *********

Fiu_refseq      CAAGTGATTAAAGATCAGGGTGCAACCAACCTTACCGACGCGCTCAAAAACGTCCCCGGC   300
70233           CAAGTGATTAAAGATCAGGGCGCAACCAACCTTACCGACGCACTCAAAAACGTCCCCGGC   300
70238           CAAGTGATTAAAGATCAGGGCGCAACCAACCTTACCGACGCGCTCAAAAACGTCCCCGGC   300
70234           CAAGTGATTAAAGATCAGGGCGCAACCAACCTTACCGACGCGCTCAAAAACGTCCCCGGC   300
70239           CAAGTGATTAAAGATCAGGGCGCAACCAACCTTACCGACGCGCTCAAAAACGTCCCCGGC   300
70242           CAAGTGATTAAAGATCAGGGCGCAACCAACCTTACCGACGCGCTCAAAAACGTCCCCGGC   300
70237           CAAGTGATTAAAGATCAGGGTGCAACCAACCTTACCGACGCGCTCAAAAACGTCCCCGGC   300
70240           CAAGTGATTAAAGATCAGGGCGCAACCAACCTTACCGACGCGCTCAAAAACGTCCCCGGC   300
                ******************** ******************** ******************

Fiu_refseq      GTGGGTGCGTTTTTTGCGGGTGAGAACGGTAACTCCACCACTGGCGACGCCATTTATATG   360
70233           GTGGGTGCGTTTTTTGCGGGTGAGAACGGTAACTCCACCACTGGCGACGCCATTTATATG   360
70238           GTGGGTGCGTTTTTTGCGGGTGAGAACGGTAACTCCACCACTGGCGACGCCATTTATATG   360
70234           GTGGGTGCGTTTTTTGCGGGTGAGAACGGTAACTCCACCACTGGCGACGCCATTTATATG   360
70239           GTGGGTGCGTTTTTTGCGGGTGAGAACGGTAACTCCACCACTGGCGACGCCATTTATATG   360
70242           GTGGGTGCGTTTTTTGCGGGTGAGAACGGTAACTCCACCACTGGCGACGCCATTTATATG   360
```

```
70237         GTGGGTGCGTTTTTTGCGGGTGAGAACGGTAACTCCACCACTGGCGACGCCATTTATATG   360
70240         GTGGGTAATGCCGATCAGTTAAGGATCAGTTGA---------------------------   333
              ******        *  *     ** * **   ***************************

Fiu_refseq    CGCGGTGCCGATACCTCTAACAGTATTTATATCGATGGCATTCGCGATATCGGCAGCGTC   420
70233         CGCGGTGCTGATACATCTAACAGTATTTATATTGATGGCATTCGCGATATCGGCAGCGTC   420
70238         CGTGGTGCCGATACCTCTAACAGTATTTATATTGATGGCATTCGCGATATCGGCAGCGTC   420
70234         CGTGGTGCCGATACCTCTAACAGTATTTATATTGATGGCATTCGCGATATCGGCAGCGTC   420
70239         CGCGGTGCTGATACCTCTAACAGTATTTATATTGATGGCATTCGCGATATCGGCAGCGTC   420
70242         CGCGGTGCTGATACCTCTAACAGTATTTATATTGATGGCATTCGCGATATCGGCAGCGTC   420
70237         CGCGGTGCCGATACCTCTAACAGTATTTATATCGATGGCATTCGCGATATCGGCAGCGTC   420
70240
              ** ***** ***** ***************** ***************************

Fiu_refseq    TCGCGCGACACCTTCAATACCGAGCAGGTCGAAGTAATTAAAGGGCCGTCCGGCACCGAC   480
70233         TCGCGCGACACCTTCAATACCGAGCAGGTCGAAGTGATTAAAGGGCCTTCCGGCACCGAC   480
70238         TCGCGCGACACCTTCAATACCGAGCAGGTAGAAGTGATTAAAGGGCCGTCCGGCACCGAC   480
70234         TCGCGCGACACCTTCAATACCGAGCAGGTCGAAGTGATTAAAGGGCCGTCCGGCACCGAC   480
70239         TCGCGCGACACCTTCAATACTGAGCAGGTCGAAGTGATTAAAGGGCCTTCCGGCACCGAC   480
70242         TCGCGCGACACCTTCAATACTGAGCAGGTCGAAGTGATTAAAGGGCCTTCCGGCACCGAC   480
70237         TCGCGCGACACCTTCAATACCGAGCAGGTCGAAGTAATTAAAGGGCCGTCCGGCACCGAC   480
70240
              ******************** ******** ***** *********** ************

Fiu_refseq    TACGGGCGCAGCGCACCGACAGGCTCGATCAATATGATCAGCAAACAGCCGCGCAATGAT   540
70233         TACGGGCGCAGCGCGCCGACGGGCTCGATCAATATGATCAGCAAGCAGCCGCGCAATGAT   540
70238         TACGGGCGCAGCGCGCCGACGGGCTCGATCAATATGATCAGCAAGCAGCCGCGCAATGAT   540
70234         TACGGGCGCAGCGCACCGACAGGCTCGATCAATATGATCAGCAAGCAGCCGCGCAATGAT   540
70239         TACGGGCGCAGCGCGCCGACGGGCTCGATCAATATGATCAGCAAGCAGCCGCGCAATGAT   540
70242         TACGGGCGCAGCGCGCCGACGGGCTCGATCAATATGATCAGCAAGCAGCCGCGCAATGAT   540
70237         TACGGGCGCAGCGCGCCGACAGGCTCGATCAATATGATCAGCAAACAGCCGCGCAATGAT   540
70240
              ************** ***** *********************** ***************

Fiu_refseq    TCCGGCATTGACGCCTCCGCCAGTATTGGCAGCGCCTGGTTCCGCCGCGGCACGCTGGAC   600
70233         TCCGGCATTGACGCCTCCGCCAGTATTGGCAGCGCCTGGTTCCGCCGCGGCACGCTGGAC   600
70238         TCCGGCATTGACGCCTCCGCGAGTATTGGCAGCGCCTGGTTCCGCCGCGGCACGCTGGAC   600
70234         TCCGGCATTGACGCCTCCGCCAGTATTGGCAGCGCCTGGTTCCGCCGCGGCACGCTGGAC   600
70239         TCCGGCATTGACGCCTCCGCCAGTATTGGCAGCGCCTGGTTCCGCCGCGGCACGCTGGAC   600
70242         TCCGGCATTGACGCCTCCGCCAGTATTGGCAGCGCCTGGTTCCGCCGCGGCACGCTGGAC   600
70237         TCCGGCATTGACGCCTCCGCCAGTATTGGCAGCGCCTAGTTCCGCCGCGGCACGCTGGAC   600
70240
              ******************** **************** **********************

Fiu_refseq    GTCAATCAGGTCATTGGTGATACCACTGCGGTGCGCCTGAATGTAATGGGCGAAAAAACG   660
70233         GTCAATCAGGTCATTGGTGATACCACCGCGGTGCGCCTGAATGTGATGGGCGAAAAAACG   660
70238         GTCAATCAGGTCATTGGTGATACCACTGCGGTGCGCCTGAACGTGATGGGCGAAAAAACG   660
70234         GTCAATCAGGTCATTGGTGATACCACCGCGGTGCGCCTGAATGTAATGGGCGAAAAAACG   660
70239         GTCAATCAGGTCATTGGTGATACCACTGCGGTGCGCCTGAATGTGATGGGCGAAAAAACG   660
70242         GTCAATCAGGTCATTGGTGATACCACTGCGGTGCGCCTGAATGTGATGGGCGAAAAAACG   660
70237         GTCAATCAGGTCATTGGTGATACCACTGCGGTGCGCCTGAATGTAATGGGCGAAAAAACG   660
70240
              ************************** ************** ** ***************

Fiu_refseq    CACGATGCCGGACGCGACAAAGTCAAAAATGAGCGTTACGGCGTCGCCCCTTCTGTCGTT   720
70233         CACGATGCCGGACGCGACAAAGTCAAAAATGAGCGTTACGGCATCGCCCCTTCTGTCGCT   720
70238         CACGATGCCGGACGCGACAAAGTCAAAAATGAGCGTTACGGCGTCGCCCCTTCTGTCGCT   720
70234         CACGATGCCGGACGCGACAAAGTCAAAAATGAGCGTTACGGCGTCGCCCCTTCTGTCGCT   720
70239         CACGATGCCGGACGCGACAAAGTCAAAAATGAGCGTTACGGCGTCGCCCCTTCTATCGCT   720
70242         CACGATGCCGGACGCGACAAAGTCAAAAATGAGCGTTACGGCGTCGCCCCTTCTATCGCT   720
70237         CACGATGCCGGACGCGACAAAGTCAAAAATGAGCGTTACGGCGTCGCCCCTTCTGTCGCT   720
70240
```

```
                   ****************************************** *********** *** *

Fiu_refseq         TTTGGCCTTGGTACAGCGAATCGTTTGTATCTTAATTATCTGCATGTCACCCAGCACAAC   780
70233              TTTGGCCTTGGTACAGCGAATCGTTTGTATCTTAATTATCTGCATGTCACCCAGCACAAC   780
70238              TTTGGCCTTGGTACAGCGAATCGTTTGTATCTTAATTATCTGCATGTCACCCAGCACAAC   780
70234              TTTGGCCTTGGTACAGCGAATCGTTTGTATCTTAATTATCTGCATGTCACCCAGCACAAC   780
70239              TTTGGCCTTGGTACAGCGAATCGTTTGTATCTTAATTATCTGCATGTCACCCAGCACAAC   780
70242              TTTGGCCTTGGTACAGCGAATCGTTTGTATCTTAATTATCTGCATGTCACCCAGCACAAC   780
70237              TTTGGCCTTGGTACAGCGAATCGTTTGTATCTTAATTATCTGCATGTCACCCAGCACAAC   780
70240
                   ************************************************************

Fiu_refseq         ACGCCAGACGGCGGCATTCCGACCATCGGTTTACCGGGTTATTCTGCCCCATCTGCGGGA   840
70233              ACGCCAGACGGCGGCATTCCAACCATCGGTTTGCCGGGCTATTCTGCCCCATCTGCAGGA   840
70238              ACGCCAGACGGCGGCATTCCGACCATCGGTTTGCCGGGCTATTCTGCCCCATCTGCGGGA   840
70234              ACGCCAGACGGCGGCATTCCGACCATCGGTTTGCCGGGCTATTCTGCCCCATCTGCGGGA   840
70239              ACGCCAGACGGCGGCATTCCGACCATCGGTTTGCCGGGTTATTCTGCCCCATCTGCGGGA   840
70242              ACGCCAGACGGCGGCATTCCGACCATCGGTTTGCCGGGTTATTCTGCCCCATCTGCGGGA   840
70237              ACGCCAGACGGCGGCATTCCGACTATCGGTTTACCGGGTTATTCTGCCCCATCTGCGGGA   840
70240
                   ******************** *********** ***** ***************** ***

Fiu_refseq         ACAGCAGCCCTGAATCATTCCGGAAAAGTTGATACTCATAACTTTTACGGCACAGATTCC   900
70233              ACGGCGACCCTGAATCATTCCGGAAAAGTTGATACGCATAACTTTTACGGCACGGATTCC   900
70238              ACAGCGGCCCTGAATCATTCCGGAAAAGTTGATACTCATAACTTTTACGGCACGGATTCC   900
70234              ACGGCGGCCCTGAATCATTCCGGAAAAGTTGATACTCATAACTTTTACGGCACGGATTCC   900
70239              ACAGCGGCTCTGAATCATTCCGGAAAAGTTGATACTCATAACTTTTACGGCACGGATTCC   900
70242              ACAGCGGCTCTGAATCATTCCGGAAAAGTTGATACTCATAACTTTTACGGCACGGATTCC   900
70237              ACAGCGGCTCTGAATCATTCCGGAAAAGTTGATACTCATAACTTTTACGGCACGGATTCC   900
70240
                   ** **  * ************************** ***************** ******

Fiu_refseq         GATTACGACGATTCGACCACCGACACCGCTACCATGCGTTTTGAGCACGACATCAACGAT   960
70233              GATTACGACGATTCGACCACCGACACCGCTACCATGCGTTTTGAGCACGACATCAACGAT   960
70238              GATTACGACGATTCGACCACCGACACCGCTACCATGCGTTTTGAGCACGACATCAACGAT   960
70234              GATTACGACGATTCGACCACCGACACCGCCACCATGCGTTTTGAGCACGACATCAACGAT   960
70239              GATTACGACGATTCGACCACCGACACCGCCACCATGCGTTTTGAGCACGACATCAACGAT   960
70242              GATTACGACGATTCGACCACCGACACCGCCACCATGCGTTTTGAGCACGACATCAACGAT   960
70237              GATTACGACGATTCGACCACCGACACCGCTACCATGCGTTTTGAGCACGACATCAACGAT   960
70240
                   ***************************** ******************************

Fiu_refseq         AACACCACCATTCGCAACACTACGCGTTGGTCGCGCGTAAAGCAGGATTACCTGATGACG   1020
70233              AACACCACCATTCGCAACACTACCCATTGGTCGCGCGTGAAGCAGGATTACCTGATGACG   1020
70238              AACACCACCATTCGCAATACTACCCGTTGGTCGCGCGTGAAGCAGGATTACCTGATGACG   1020
70234              AACACCACCATTCGCAATACTACCCGTTGGTCGCGCGTAAAGCAGGATTACCTGATGACG   1020
70239              AACACCACCATTCGCAATACTACCCGTTGGTCGCGCGTAAAGCAGGATTACCTGATGACG   1020
70242              AACACCACCATTCGCAATACTACCCGTTGGTCGCGCGTAAAGCAGGATTACCTGATGACG   1020
70237              AACACCACCATTCGCAATACTACCCGTTGGTCGCGCGTGAAGCAGGATTACCTGATGACG   1020
70240
                   ***************** ***** * ************ *********************

Fiu_refseq         GCGATTATGGGTGGAGCGTCGAATATTACCCAGCCCACCAGCGATGTGAATAGCTGGACG   1080
70233              GCGATTATGGGTGGGGCGTCGAATATTACCCAGCCCACCAGCGATGTGAATAGCTGGACC   1080
70238              GCGATTATGGGCGGGGCGTCGAATATTACCCAGCCCACCAGCGATGTGAATAGCTGGACC   1080
70234              GCGATTATGGGCGGGGCGTCGAATATTACTCAGCCCACCAGCGATGTGAATAGCTGGACC   1080
70239              GCGATTATGGGCGGGGCGTCGAATATTACTCAGCCCACCAGCGATGTGAATAGCTGGACC   1080
70242              GCGATTATGGGCGGGGCGTCGAATATTACTCAGCCCACCAGCGATGTGAATAGCTGGACC   1080
70237              GCGATTATGGGCGGGGCGTCGAATATTACCCAGCCCACCAGCGATGTGAATAGCTGGACC   1080
70240
                   *********** ** ************** *****************************
```

```
Fiu_refseq      TGGTCACGCACGGCGAATACCAAAGATGTGAGTAACAAAATTCTCACCAACCAGACCAAC    1140
70233           TGGTCACGCACGGCGAATACTAAAGATGTGAGTAATAAAATTCTCACCAACCAGACCAAC    1140
70238           TGGTCACGCACGGCGAATACTAAAGATGTGAGTAATAAAATTCTCACCAACCAGACCAAC    1140
70234           TGGTCACGCACGGCGAATACCAAAGATGTGAGTAATAAAATTCTCACCAACCAGACCAAC    1140
70239           TGGTCACGCACGGCGAATACCAAAGATGTGAGTAATAAAATTCTCACCAACCAGACCAAC    1140
70242           TGGTCACGCACGGCGAATACCAAAGATGTGAGTAATAAAATTCTCACCAACCAGACCAAC    1140
70237           TGGTCACGCACGGCGAATACTAAAGATGTGAGTAATAAAATTCTCACCAACCAGACCAAC    1140
70240
                ******************** ************** ************************

Fiu_refseq      CTGACCTCGACGTTCTATACCGGCGCTATCGGTCATGATCTCAGTACCGGCGTGGAATTT    1200
70233           CTGACCTCGACGTTCTATACCGCTTCTATCGGTCATGATGTCAGTACCGGCGTGGAATTT    1200
70238           CTGACCTCGACGTTCTATACCGGTTCTATCGGTCATGATGTCAGTACCGGCGTGGAATTT    1200
70234           CTGACCTCGACGTTCTATACCGGTTCTATCGGTCATGATGTCAGTACCGGCGTGGAATTT    1200
70239           CTGACCTCGACGTTCTATACCGGTTCTATCGGTCATGATGTCACTACCGGCGTGGAATTT    1200
70242           CTGACCTCGACGTTCTATACCGGTTCTATCGGTCATGATGTCACTACCGGCGTGGAATTT    1200
70237           CTGACCTCGACGTTCTATACCGGCGCTATCGGTCATGATGTCAGTACCGGCGTGGAATTT    1200
70240
                **********************   ************** *** ****************

Fiu_refseq      ACCCGCGAAACACAGACTAACTATGGCGTTAATCCGGTGACGTTACCGGCGGTAAATATT    1260
70233           ACCCGTGAAACGCAGACTAACTACGGCGTTAATCCGGTGACGTTACCTGCGGTAAATATT    1260
70238           ACCCGTGAAACGCAGACTAACTACGGCGTTAATCCGGTGACGTTACCTGCGGTAAATATT    1260
70234           ACCCGTGAAACGCAGACTAACTACGGCGTTAATCCGGTGACGTTACCCGCGGTAAATATT    1260
70239           ACCCGTGAAACGCAGACTAACTACGGCGTTAATCCGGTGACGTTACCCGCGGTAAATATT    1260
70242           ACCCGTGAAACGCAGACTAACTACGGCGTTAATCCGGTGACGTTACCCGCGGTAAATATT    1260
70237           ACCCGTGAAACGCAGACTAACTACGGCGTTAATCCGGTGACGTTACCTGCGGTAAATATT    1260
70240
                ***** ***** *********** *********************** ************

Fiu_refseq      TATCATCCTGACAGCAGCATTCATCCCGGCGGCCTGACGCGCAACGGCGCAAACGCCAAT    1320
70233           TATCATCCTGACAGCAGCATTCATCCCGGCGGCCTGACGCGCAACGGCGCAAACGCCAAT    1320
70238           TATCATCCTGACAGCAGCATTCATCCCGGCGGCCTGACGCGCAACGGCGCAAACGCCAAT    1320
70234           TATCATCCTGACAGCAGCATTCATCCCGGCGGCCTGACGCGCAACGGCGCAAACGCCAAT    1320
70239           TATCATCCTGACAGCAGCATTCATCCCGGCGGCCTGACGCGCAACGGCGCAAACGCCAAT    1320
70242           TATCATCCTGACAGCAGCATTCATCCCGGCGGCCTGACGCGCAACGGCGCAAACGCCAAT    1320
70237           TATCATCCTGACAGCAGCATTCATCCCGGCGGCCTGACGCGCAACGGCGCAAACGCCAAT    1320
70240
                ************************************************************

Fiu_refseq      GGTCAGACGGATACCTTCGCAATTTATGCCTTCGATACGCTGCAAATCACCCGTGATTTT    1380
70233           GGTCAGACGGATACCTTCGCAATTTACGCCTTCGATACGCTGCAAATCACCCGTGATTTT    1380
70238           GGTCAGACGGATACCTTCGCAATTTACGCCTTCGATACGCTGCAAATCACCCGTGATTTT    1380
70234           GGTCAGACGGATACCTTCGCAATTTACGCCTTTGATACGCTGCAAATCACCCGTGATTTT    1380
70239           GGTCAGACGGATACCTTCGCAATTTACGCCTTCGATACGCTGCAAATCACCCGTGATTTT    1380
70242           GGTCAGACGGATACCTTCGCAATTTACGCCTTCGATACGCTGCAAATCACCCGTGATTTT    1380
70237           GGTCAGACGGATACCTTCGCAATTTACGCCTTCGATACGCTGCAAATCACCCGTGATTTT    1380
70240
                ************************** ***** ***************************

Fiu_refseq      GAGCTGAACGGCGGGATCCGTCTGGATAATTATCATACTGAATATGACAGTGCTACCGCC    1440
70233           GAGCTGAACGGCGGGATCCGTCTGGATAATTATCATACTGAATATGACAGTGCCACCGCC    1440
70238           GAGCTGAACGGCGGGATCCGTCTGGATAATTATCATACTGAATATGACAGTGCCACCGCC    1440
70234           GAGCTGAACGGCGGGATCCGTCTGGATAATTATCATACTGAATATGACAGTGCCACCGCC    1440
70239           GAGCTGAACGGCGGGATCCGTCTGGATAATTATCATACTGAATATGACAGTGCCACCGCC    1440
70242           GAGCTGAACGGCGGGATCCGTCTGGATAATTATCATACTGAATATGACAGTGCCACCGCC    1440
70237           GAGCTGAACGGCGGGATCCGTCTGGATAATTATCATACTGAATATGACAGTGCCACCGCC    1440
70240
                ***************************************************** ******

Fiu_refseq      TGCGGCGGCAGCGGGCGCGGTGCCATCACCTGCCCTGCTGGCGTGGCAAAAGGTTCTCCG    1500
70233           TGCGGCGGCAGCGGACGCGGTGCCATCACCTGCCCAGCTGGTGTGGCAAAAGGTTCTCCG    1500
```

```
70238              TGCGGCGGCAGCGGACGCGGTGCCATCACCTGCCCTGCTGGCGTGGCAAAAGGTTCTCCG    1500
70234              TGCGGCGGCAGCGGACGCGGTGCCATCACCTGCCCAACTGGTGTGGCAAAAGGTTCTCCG    1500
70239              TGCGGCGGCAGCGGACGCGGTGCCATCACCTGCCCAACTGGTGTGGCAAAAGGTTCTCCG    1500
70242              TGCGGCGGCAGCGGACGCGGTGCCATCACCTGCCCAACTGGTGTGGCAAAAGGTTCTCCG    1500
70237              TGCGGCGGCAGCGGACGCGGTGCCATCACCTGCCCTGCTGGCGTGGCAAAAGGTTCTCCG    1500
70240
                   ************** ********************  **** ******************

Fiu_refseq         GTCACCACCGTCGACACCGCCAAGTCGGGCAATCTGGTGAACTGGAAAGCCGGGGCGCTG    1560
70233              GTCACCACCGTCGACACCGCCAAGTCGGGCAATCTGGTGAACTGGAAAGCCGGGGCGCTG    1560
70238              GTCACCACCGTCGATACCGCCAAGTCGGGCAATCTGGTGAACTGGAAAGCCGGGGCGCTG    1560
70234              GTCACCACCGTCGACACCGCCAAGTCGGGCAATCTGATGAACTGGAAAGCCGGGGCGCTG    1560
70239              GTCACCACCGTCGACACCGCCAAGTCGGGCAATCTGGTGAACTGGAAAGCCGGGGCGCTG    1560
70242              GTCACCACCGTCGACACCGCCAAGTCGGGCAATCTGGTGAACTGGAAAGCCGGGGCGCTG    1560
70237              GTCACCACCGTCGATACCGCCAAGTCGGGCAATCTGGTGAACTGGAAAGCCGGGGCGCTG    1560
70240
                   ************** ******************** ************************

Fiu_refseq         TATCACCTGACGGAAAACGGCAATGTCTATATTAACTATGCCGTTTCCCAGCAGCCGCCG    1620
70233              TATCACCTGACGGAAAATGGCAATGTCTATATTAACTATGCCGTTTCCCAGCAGCCTCCG    1620
70238              TATCACCTGACGGAAAACGGCAATGTCTATATTAACTATGCCGTTTCCCAGCAGCCTCCG    1620
70234              TATCACCTGACGGAAAACGGCAATGTCTATATTAACTATGCCGTTTCCCAGCAGCCTCCG    1620
70239              TATCACCTGACGGAAAACGGTAATGTCTATATTAACTATGCCGTTTCCCAGCAGCCTCCG    1620
70242              TATCACCTGACGGAAAACGGTAATGTCTATATTAACTATGCCGTTTCCCAGCAGCCTCCG    1620
70237              TATCACCTGACGGAAAACGGCAATGTCTATATTAACTATGCCGTTTCCCAGCAGCCTCCG    1620
70240
                   ***************** ** *********************************** ***

Fiu_refseq         GGCGGCAACAACTTCGCCCTTGCGCAGTCTGGCAGCGGTAACAGTGCCAACCGCACCGAT    1680
70233              GGCGGCAACAACTTCGCCCTTGCGCAGTCTGGCAGCGGTAACAGTGCCAACCGCACCGAT    1680
70238              GGCGGCAACAACTTCGCCCTTGCGCAGTCTGGCAGCGGTAACAGTGCCAACCGCACCGAT    1680
70234              GGCGGCAACAACTTCGCCCTTGCGCAGTCTGGCAGCGGTAACAGTGCCAACCGCACCGAT    1680
70239              GGCGGCAACAACTTCGCCCTTGCGCAGTCTGGCAGCGGTAACAGTGCCAACCGCACCGAT    1680
70242              GGCGGCAACAACTTCGCCCTTGCGCAGTCTGGCAGCGGTAACAGTGCCAACCGCACCGAT    1680
70237              GGCGGCAACAACTTCGCCCTTGCGCAGTCTGGCAGCGGTAACAGTGCCAACCGCACCGAT    1680
70240
                   ************************************************************

Fiu_refseq         TTTAAACCACAAAAAGCCAACACCAGCGAGATTGGCACCAAATGGCAGGTTCTGGATAAA    1740
70233              TTTAAACCGCAAAAAGCCAACACCAGCGAGATTGGCACCAAATGGCAGGTTCTGGATAAA    1740
70238              TTTAAACCGCAAAAAGCCAACACCAGCGAGATTGGCACCAAATGGCAGGTTCTGGATAAA    1740
70234              TTTAAACCGCAAAAAGCCAACACCAGCGAGATTGGCACCAAATGGCAGGTTCTGGATAAA    1740
70239              TTTAAACCGCAAAAAGCCAACACCAGCGAGATTGGCACCAAATGGCAGGTTCTGGATAAA    1740
70242              TTTAAACCGCAAAAAGCCAACACCAGCGAGATTGGCACCAAATGGCAGGTTCTGGATAAA    1740
70237              TTTAAACCGCAAAAAGCCAACACCAGCGAGATTGGCACCAAATGGCAGGTTCTGGATAAA    1740
70240
                   ******** ***************************************************

Fiu_refseq         CGCCTGCTACTTACCGCCGCGCTGTTCCGTACTGATATTGAAAATGAAGTTGAGCAAAAT    1800
70233              CGCCTGTTGCTCACCGCCGCGCTGTTCCGTACTGATATCGAAAATGAAGTTGAGCAAAAT    1800
70238              CGCCTGTTGCTCACCGCCGCGCTGTTCCGTACTGATATCGAAAATGAAGTTGAGCAAAAT    1800
70234              CGTCTGTTGCTCACCGCCGCGCTGTTCCGCACTGATATCGAAAATGAAGTTGAGCAAAAT    1800
70239              CGCCTGTTGCTCACCGCCGCGCTATTCCGTACTGATATCGAAAATGAAGTTGAGCAAAAT    1800
70242              CGCCTGTTGCTCACCGCCGCGCTATTCCGTACTGATATCGAAAATGAAGTTGAGCAAAAT    1800
70237              CGCCTGTTGCTCACCGCCGCGCTGTTCCGTACTGATATCGAAAATGAAGTTGAGCAAAAT    1800
70240
                   ** *** * ** *********** ***** ******** *********************

Fiu_refseq         GATGACGGGACTTATTCGCAATATGGTAAGAAACGTGTCGAAGGCTACGAGATATCCGTG    1860
70233              GATGACGGAACTTACTCGCAATATGGTAAGAAACGCGTCGAAGGCTATGAGATATCCGTG    1860
70238              GATGACGGAACTTACTCGCAATACGGTAAGAAACGCGTCGAAGGCTATGAGATATCCGTG    1860
70234              GATGACGGAACTTACTCGCAATACGGTAAGAAACGCGTCGAAGGCTATGAGATATCCGTG    1860
```

```
70239         GATGACGGAACTTACTCGCAATACGGTAAGAAACGTGTCGAAGGCTATGAGATATCCGTG   1860
70242         GATGACGGAACTTACTCGCAATACGGTAAGAAACGTGTCGAAGGCTATGAGATATCCGTG   1860
70237         GATGACGGAACTTACTCGCAATACGGTAAGAAACGCGTCGAAGGCTATGAGATATCCGTG   1860
70240
              ******** ***** ******** *********** *********** ************

Fiu_refseq    GCCGGGAATATCACCCCCGCATGGCAGATGATTGGCGGCTATACCCAGCAAAAAGCAACT   1920
70233         GCCGGGAATATCAGTCCCGCGTGGCAGGTGATTGGCGGCTATACCCAGCAAAAAGCAACC   1920
70238         GCCGGGAATATCACTCCCGCGTGGCAGGTGATTGGCGGCTATACCCAGCAAAAAGCAACC   1920
70234         GCCGGGAATATCACTCCCGCGTGGCAGGTGATTGGCGGCTATACCCAGCAAAAAGCAACC   1920
70239         GCCGGGAATATCACTCCCGCGTGGCAGGTGATTGGCGGCTATACCCAGCAAAAAGCAACC   1920
70242         GCCGGGAATATCACTCCCGCGTGGCAGGTGATTGGCGGCTATACCCAGCAAAAAGCAACC   1920
70237         GCCGGGAATATCACTCCCGCGTGGCAGGTGATTGGCGGCTATACCCAGCAAAAAGCAACC   1920
70240
              *************  ***** ****** *******************************

Fiu_refseq    ATCAAAAACGGCAAAGATGTTGCCCAGGATGGTTCCTCATCGCTGCCGTATACCCCGGAA   1980
70233         ATCAAAAACGGCAAAGATGTTGCCCAGGATGGTTCCTCATCGCTGCCCTATACCCCGGAG   1980
70238         ATCAAAAACGGCAAAGATGTTGCCCAGGATGGTTCCTCATCGCTGCCGTATACCCCGGAG   1980
70234         ATCAAAAACGGCAAAGATGTTGCCCAGGATGGTTCCTCATCGCTGCCGTATACCCCGGAG   1980
70239         ATCAAAAACGGCAAAGATGTTGCCCAGGATGGTTCCTCATCGCTGCCGTATACCCCGGAG   1980
70242         ATCAAAAACGGCAAAGATGTTGCCCAGGATGGTTCCTCATCGCTGCCGTATACCCCGGAG   1980
70237         ATCAAAAACGGCAAAGATGTTGCCCAGGATGGTTCCTCATCGCTGCCGTATACCCCGGAG   1980
70240
              *********************************************** ***********

Fiu_refseq    CACGCCTTCACCTTATGGAGCCAGTATCAGGCAACCGATGATATTTCTGTTGGTGCGGGC   2040
70233         CACGCCTTTACCTTATGGAGCCAGTATCAGGCAACCGATGATATTTCCGTTGGTGCGGGC   2040
70238         CACGCCTTTACCTTGTGGAGCCAGTATCAGGCAACCGATGATATTTCCGTTGGTGCGGGC   2040
70234         CACGCCTTCACCTTATGGAGCCAATATCAGGCAACCGACGATATCTCTGTTGGCGCGGGC   2040
70239         CACGCCTTCACCTTATGGAGCCAGTATCAGGCAACCGATGATATCTCTGTTGGCGCGGGC   2040
70242         CACGCCTTCACCTTATGGAGCCAGTATCAGGCAACCGATGATATCTCTGTTGGCGCGGGC   2040
70237         CACGCCTTTACCTTATGGAGCCAGTATCAGGCAACCGATGATATTTCCGTTGGTGCGGGC   2040
70240
              ******** ***** ******** ************** ***** ** ***** ******

Fiu_refseq    GCACGGTATATCGGCAGTATGCATAAAGGTTCAGACGGCGCGGTGGGGACGCCAGCATTT   2100
70233         GCACGGTATATCGGCAGTATGCATAAAGGTTCAGACGGCGCGGTGGGAACGCCAGCATTT   2100
70238         GCACGCTATATCGGCAGTATGCATAAAGGTTCTGACGGCGCGGTGGGAACGCCAGCGTTT   2100
70234         GCACGCTATATCGGCAGTATGCATAAAGGTTCAGACGGCGCGGTGGGAACGCCAGCGTTT   2100
70239         GCACGCTATATCGGCAGTATGCATAAAGGTTCAGACGGCGCGGTGGGAACGCCAGCGTTT   2100
70242         GCACGCTATATCGGCAGTATGCATAAAGGTTCAGACGGCGCGGTGGGAACGCCAGCGTTT   2100
70237         GCACGCTATATCGGCAGTATGCATAAAGGTTCTGACGGCGCGGTGGGAACGCCAGCGTTT   2100
70240
              ***** ************************** ************** ******** ***

Fiu_refseq    ACCGAAGGTTATTGGGTCGCCGACGCCAAACTGGGGTATCGGGTTAATCGCAATCTCGAC   2160
70233         ACTGAAGGTTACTGGGTCGCCGACGCCAAACTGGGGTATCGGGTTAATCGCAATCTCGAC   2160
70238         ACCGAAGGTTATTGGGTCGCCGACGCTAAACTGGGGTATCGGGTTAATCGCAATCTCGAC   2160
70234         ACCGAAGGTTACTGGGTCGCCGATGCCAAACTGGGGTATCGAGTTAATCGCAATCTCGAC   2160
70239         ACCGAAGGTTACTGGGTCGCCGATGCCAAACTGGGGTATCGGGTTAATCGCAATCTCGAC   2160
70242         ACCGAAGGTTACTGGGTCGCCGATGCCAAACTGGGGTATCGGGTTAATCGCAATCTCGAC   2160
70237         ACCGAAGGTTATTGGGTCGCCGACGCTAAACTGGGGTATCGGGTTAATCGCAATCTCGAC   2160
70240
              ** ******** *********** ** ************** ******************

Fiu_refseq    TTCCAGCTAAACGTCTACAACCTGTTTGATACCGATTACGTCGCCTCAATCAATAAGAGC   2220
70233         TTCCAGCTAAACGTCTACAACCTGTTTGATACCGATTACGTCGCCTCAATCAACAAGAGC   2220
70238         TTCCAGCTAAACGTCTACAACCTGTTTGATACCGATTACGTCGCCTCAATCAACAAGAGC   2220
70234         TTCCAGCTAAACGTTTACAACCTGTTTGATACCGATTACGTCGCCTCAATCAACAAGAGC   2220
70239         TTCCAGCTAAACGTTTACAACCTGTTTGATACCGATTACGTCGCCTCAATCAACAAGAGC   2220
70242         TTCCAGCTAAACGTTTACAACCTGTTTGATACCGATTACGTCGCCTCAATCAACAAGAGC   2220
```

```
70237           TTCCAGCTAAACGTCTACAACCTGTTTGATACCGATTACGTCGCCTCAATCAACAAGAGC    2220
70240
                ************** ************************************* *****


Fiu_refseq      GGCTACCGTTATCACCCGGGCGAGCCAAGAACCTTCTTGCTCACAGCTAATATGCATTTC    2280
70233           GGCTACCGTTATCACCCGGGCGAGCCAAGAACCTTCTTGCTCACAGCCAATATGCATTTC    2280
70238           GGCTACCGTTATCACCCGGGCGAGCCAAGAACCTTCCTGCTCACGGCCAATATGCATTTC    2280
70234           GGCTACCGTTATCACCCGGGCGAGCCAAGAACCTTCTTGCTCACAGCCAATATGCATTTC    2280
70239           GGCTACCGTTATCACCCGGGCGAGCCAAGAACCTTCTTGCTCACAGCCAATATGCATTTC    2280
70242           GGCTACCGTTATCACCCGGGCGAGCCAAGAACCTTCTTGCTCACAGCCAATATGCATTTC    2280
70237           GGCTACCGTTATCACCCGGGCGAGCCAAGAACCTTCCTGCTCACGGCCAATATGCATTTC    2280
70240
                ************************************ ******* ** ***********


Fiu_refseq      TGA     2283
70233           TGA     2283
70238           TGA     2283
70234           TGA     2283
70239           TGA     2283
70242           TGA     2283
70237           TGA     2283
70240
```
